# Comparative Benchmark of Sampling-Based and DRL Motion Planning Methods for Industrial Robotic Arms

**DOI:** 10.3390/s25175282

**Published:** 2025-08-25

**Authors:** Ignacio Fidalgo Astorquia, Guillermo Villate-Castillo, Alberto Tellaeche, Juan-Ignacio Vazquez

**Affiliations:** 1 Department of Computing, Electronics and Communication Technologies, University of Deusto, Avenida de las Universidades 24, 48007 Bilbao, Spain; alberto.tellaeche@deusto.es (A.T.); ivazquez@deusto.es (J.-I.V.); 2 TECNALIA, Basque Research and Technology Alliance (BRTA), 48160 Derio, Spain

**Keywords:** deep reinforcement learning (DRL), motion planning, industrial robotics, sampling-based planners, Open Motion Planning Library (OMPL), hybrid motion planning, curriculum learning

## Abstract

This study presents a comprehensive comparison between classical sampling-based motion planners from the Open Motion Planning Library (OMPL) and a learning-based planner based on Soft Actor–Critic (SAC) for motion planning in industrial robotic arms. Using a UR3e robot equipped with an RG2 gripper, we constructed a large-scale dataset of over 100,000 collision-free trajectories generated with MoveIt-integrated OMPL planners. These trajectories were used to train a DRL agent via curriculum learning and expert demonstrations. Both approaches were evaluated on key metrics such as planning time, success rate, and trajectory smoothness. Results show that the DRL-based planner achieves higher success rates and significantly lower planning times, producing more compact and deterministic trajectories. Time-optimal parameterization using TOPPRA ensured the dynamic feasibility of all trajectories. While classical planners retain advantages in zero-shot adaptability and environmental generality, our findings highlight the potential of DRL for real-time and high-throughput motion planning in industrial contexts. This work provides practical insights into the trade-offs between traditional and learning-based planning paradigms, paving the way for hybrid architectures that combine their strengths.

## 1. Introduction

Motion planning plays a foundational role in industrial robotics, enabling the autonomous execution of complex tasks such as pick-and-place, assembly, welding, and inspection. Traditionally, trajectory generation in industrial environments has been treated as a deterministic problem: robots are programmed to follow predefined joint trajectories learned through teaching or pendant-based demonstration [1]. Once recorded, these trajectories are stored and reused with minimal variation, providing cycle–time consistency and satisfying safety and certification requirements. This approach is well-suited for structured, repetitive operations in static workcells [2,3].

However, modern manufacturing trends increasingly demand greater flexibility, adaptability, and autonomy [4]. Applications such as collaborative robotics, bin picking, machine tending, and dynamic part assembly require robots to plan motions in partially unknown or variable environments. In such scenarios, predefined trajectories may no longer suffice, and the ability to compute feasible motions on-the-fly becomes essential. As a result, general-purpose motion planning has become a key capability in industrial robotic systems.

Current solutions to this challenge rely predominantly on two families of motion planning algorithms. Optimization-based planners such as Covariant Hamiltonian Optimization for Motion Planning (CHOMP) [5], Stochastic Trajectory Optimization for Motion Planning (STOMP) [6], and TrajOpt [7] generate locally smooth trajectories by minimizing cost functions under dynamic and geometric constraints. Sampling-based planners, on the other hand, such as those implemented in the OMPL [8], explore the configuration space using randomized sampling and graph construction techniques. While sampling-based methods excel in exploring complex, high-dimensional spaces and are widely supported in frameworks like MoveIt [9], which itself is built on the Robot Operating System (ROS) [10], optimization-based methods are often used for fine-grained refinement of motion plans. Hybrid approaches that combine these two paradigms—using sampling for global planning and optimization for local refinement—are also gaining traction in both research and deployment.

Despite their wide applicability, these classical planners exhibit important limitations in the context of industrial automation. Their planning time is often non-deterministic, varying significantly depending on the complexity of the environment and the robot’s configuration. This variability hinders real-time decision-making and complicates integration with deterministic control pipelines. Additionally, the paths produced by sampling-based planners often require extensive post-processing to become dynamically feasible, while optimization-based planners may fail to escape local minima or require careful initialization [11,12]. These factors can impede the scalability of classical approaches for modern, responsive industrial applications.

In recent years, Deep Reinforcement Learning (DRL) has emerged as a promising alternative for industrial robot motion planning [13]. DRL enables the training of policies that directly map observed states to actions through experience, allowing robots to generate goal-directed motions in real time via inference. Once trained, DRL policies exhibit time-deterministic behavior, can generalize across a wide range of goal configurations, and often encode collision avoidance and joint limit constraints implicitly. These properties make DRL particularly attractive in flexible industrial setups where planning speed and robustness are critical.

Beyond motion planning, artificial intelligence methods are increasingly integrated into industrial robotics pipelines for tasks such as perception, inspection, and quality control. In manufacturing settings, vision-based defect detection plays a crucial role in ensuring product quality and reducing downtime. Recent advances in deep learning, such as novel convolutional neural network architectures for surface defect detection [14], demonstrate the growing potential of AI to enhance multiple stages of the production process. Although our work focuses on motion planning, these complementary advances highlight the broader trend of embedding learning-based components throughout industrial robotic systems.

Importantly, DRL can be seen as a hybrid approach, combining elements of both sampling-based and optimization-based planners, as it samples actions while using feedback to optimize a reward function. These paradigms attempt to solve the global motion planning problem, generating trajectories from arbitrary start to goal configurations without requiring local initialization. However, unlike sampling-based and optimization-based methods, DRL policies amortize the cost of planning over training and can produce motions with fixed latency—an appealing property for integration with industrial controllers.

While the literature on classical and learning-based planning is extensive, direct comparisons between DRL and sampling-based motion planners remain scarce. Most studies either evaluate classical planners in fixed-scenario benchmarks or investigate DRL agents in simulation, often with limited environment variability [15]. Furthermore, the comparisons that do exist typically do not operate under equivalent conditions, making it difficult to draw conclusions about performance trade-offs [16]. In particular, few works evaluate both approaches using a shared workspace, a common set of goal configurations, and unified performance metrics. The role of expert demonstrations from classical planners in accelerating DRL training also remains underexplored in systematic, large-scale studies [17].

In this work, we hypothesize that a DRL-based planner can outperform traditional sampling-based methods in terms of planning latency, success rate, and trajectory compactness in dynamic and flexible industrial settings—such as collaborative robotics and agile manufacturing—once sufficient training data is available, albeit at the cost of upfront computational effort for policy training. Adaptive motion planning is increasingly critical for modern factories, where robots must safely and efficiently react to changing tasks, human co-workers, and unpredictable environments.

### 1.1. Main Research and Contributions

In this study, we present a comprehensive comparative analysis of classical motion planning techniques and Deep Reinforcement Learning (DRL)-based motion planning for industrial robotic arms, specifically applied to a UR3e robot equipped with an RG2 gripper. The primary objective of this work is to evaluate the performance of a DRL-based planner, specifically a Soft Actor–Critic (SAC) [18] approach, against classical sampling-based planners integrated within the MoveIt framework, focusing on large-scale motion planning tasks.

Our key contributions are as follows:

- We constructed a large-scale dataset of over 100,000 motion planning queries generated using MoveIt-integrated OMPL planners to evaluate and compare both classical and learning-based motion planners.
- We introduced a DRL-based planner trained using curriculum learning and expert demonstrations to solve motion planning tasks. The DRL planner was evaluated on key performance metrics, including planning success rate, planning time, trajectory smoothness, and path compactness.
- We benchmarked the DRL-based planner against classical sampling-based planners, highlighting the advantages of DRL in terms of lower planning times, higher success rates, and more compact trajectories.

Our results indicate that while classical planners are still advantageous in certain contexts, DRL offers significant potential for real-time, high-throughput motion planning, particularly in industrial contexts requiring low-latency, deterministic performance.

This benchmark—conducted on a dense, uniformly sampled dataset covering the full workspace of the UR3e arm—provides new insights into the relative performance of learning-based and classical methods in terms of planning time, success rate, and trajectory quality, under consistent experimental conditions.

This work provides valuable insights into the trade-offs between traditional and learning-based motion planning paradigms, offering a foundation for the development of hybrid planning architectures that combine the strengths of both approaches.

### 1.2. Structure of the Paper

The paper is organized as follows: Section 2 provides a review of related work on existing motion planning techniques, including classical sampling-based and optimization-based methods, as well as the recent applications of DRL in motion planning. This section also highlights the gaps in comparative benchmarks and the need for systematic evaluations of both DRL and classical methods. In Section 3, the Materials and Methods outlines the experimental setup and methodology used to generate the large-scale dataset and train the DRL-based planner. It details the construction of the dataset, including inverse kinematics solutions, trajectory generation, and the motion planning pipeline. Section 4 reports the outcomes of the large-scale benchmarking, including the performance of the DRL-based planner and classical planners in terms of success rates, planning times, trajectory smoothness, and computational efficiency. Detailed comparisons and statistical analyses are provided to illustrate the relative performance of each approach. Section 5 examines the findings, emphasizing the advantages of the DRL-based approach and its limitations compared to classical methods. This section also explores potential future research directions, including the application of transfer learning and the integration of hybrid planning architectures that combine classical and learning-based methods. In Section 6, the Conclusions summarize the main findings and contributions of the paper, emphasizing the potential of DRL for real-time, high-throughput motion planning in industrial applications, and suggest avenues for future work, particularly in the areas of adaptability and scalability.

## 2. Related Work

This section reviews the two main classical approaches, sampling-based and optimization-based planners, and discusses their standardization through frameworks such as ROS and MoveIt. We highlight the rise of hybrid planning architectures that integrate these paradigms, as well as their respective limitations in industrial settings. Furthermore, we review recent trends in applying DRL to motion planning, analyzing its advantages and current challenges. Finally, we identify gaps in comparative benchmarking between classical and learning-based methods, motivating the need for standardized, large-scale evaluations such as the one proposed in this work.

### 2.1. Sampling-Based Motion Planning and Standardization Through ROS and MoveIt

Sampling-based motion planners have played a central role in the advancement of motion planning for robotic manipulators, particularly due to their effectiveness in high-dimensional and constrained environments. Algorithms such as Rapidly-exploring Random Trees (RRT) [19], Probabilistic Roadmaps (PRM) [20], and their many variants have become the standard for generating feasible paths when exact solutions are intractable. These planners construct a roadmap or search tree by incrementally sampling the configuration space and attempting to connect sampled points using local planning primitives. Their appeal lies in their probabilistic completeness, general-purpose applicability, and minimal reliance on task-specific heuristics.

It is important to distinguish between single-query and multi-query planners within this context. Single-query planners, such as RRT and its variants, are designed to solve individual planning problems efficiently by constructing a search tree from the start configuration to the goal. These planners are particularly effective in dynamic or frequently changing environments where precomputing a roadmap is impractical. On the other hand, multi-query planners, such as PRM, precompute a roadmap of the configuration space that can be reused for multiple planning queries. While this approach incurs a higher upfront computational cost, it is advantageous in static environments where multiple start and goal configurations need to be planned efficiently. The choice between single-query and multi-query planners depends heavily on the specific application requirements, including environment dynamics, computational resources, and the expected number of planning queries.

A major factor in the widespread adoption and continued evolution of these planners is their standardization and accessibility through the ROS ecosystem. Within ROS, the OMPL has emerged as the de facto standard for sampling-based motion planning. OMPL offers a comprehensive suite of planning algorithms, ranging from basic RRT and PRM to more advanced variants such as KPIECE [21], providing a clean separation between planning algorithms and robot- or environment-specific logic such as collision checking and state validity. This design allows OMPL to serve as a reusable planning core across a wide variety of robotic platforms.

Later advancements in RRT and PRM variants introduced several enhancements to improve their efficiency and applicability in complex motion planning scenarios [22]. Lazy evaluation techniques, such as LazyPRM [23] and LazyRRT, defer collision checks until absolutely necessary, significantly reducing computational overhead. Optimality-focused algorithms like RRT* and PRM* ensure asymptotic convergence to the shortest path by incorporating rewiring and cost-based heuristics. Bidirectional approaches, such as Bidirectional RRT (BiRRT) and Bidirectional PRM, simultaneously grow trees or roadmaps from both the start and goal configurations, accelerating convergence in high-dimensional spaces. Sparsity-aware methods, including Sparse-RRT and SPARS, aim to maintain a minimal yet sufficient set of samples to represent the configuration space, reducing memory usage while preserving solution quality. Additionally, heuristic-guided planners, such as Informed-RRT* [24] and BIT* [25], leverage domain-specific knowledge or cost-to-go estimates to focus exploration on promising regions, further improving planning efficiency and success rates.

The integration of OMPL within MoveIt, ROS’s principal motion planning framework for manipulators, has further elevated the role of sampling-based planners in both academic and industrial robotics. Its modularity has made MoveIt a powerful tool for industrial applications, allowing practitioners to tailor the planning pipeline to address use cases ranging from simple pick-and-place to dual-arm coordinated manipulation and dynamic reconfiguration tasks.

Numerous research efforts have demonstrated how ROS-Industrial [26], as an extension of ROS tailored for factory automation, has enabled the development of flexible and hardware-agnostic robot solutions across manufacturing sectors. For instance, the work by Malvido-Fresnillo et al. [27] presented critical extensions to MoveIt aimed at addressing real-world manufacturing needs, such as automatic tool changers, precise end-effector trajectory control, and coordinated dual-arm motion. These enhancements were validated on a dual-arm industrial manipulator, showing that even complex behaviors can be handled within the ROS-MoveIt stack when extended appropriately.

Similarly, Martinez et al. [28] documented the deployment of a dual-arm Motoman robot using ROS-Industrial in a dynamic research-driven industrial environment. Their work highlighted the practicality of configuring and controlling complex manipulators in ROS, and emphasized how ROS-Industrial lowers the barrier for developing advanced applications in academic and industrial collaborations.

Beyond static manufacturing environments, ROS has also been applied in cloud-distributed industrial tasks. Rahimi et al. [29] implemented an ROS-based system for surface blending using distributed computing over a wide-area network. Their setup included 3D scanning, segmentation, and path planning distributed across remote servers, demonstrating how ROS can support scalable, data-intensive tasks with real-time requirements in production settings.

More recently, researchers have leveraged ROS to develop hybrid platforms that combine mobile and dual-arm manipulators. Xu et al. [30] presented a mobile dual-arm system simulated and tested in ROS, demonstrating coordinated coupling behaviors for manipulation in logistics scenarios. Similarly, Li et al. [31] implemented a full pick-and-place pipeline using ROS-I and Gazebo in a conveyor-based industrial sorting system, showing how ROS’s perception, planning, and control packages can be integrated into tightly constrained cycle-time-sensitive tasks.

Collectively, these applications underline the growing maturity of ROS and MoveIt in industrial contexts. They demonstrate that, with appropriate extensions, these tools are not only viable for complex, sensor-integrated applications but can also support real-time performance constraints critical in manufacturing. Their continued evolution and open-source nature ensure that innovations in motion planning algorithms and system architectures remain accessible, reproducible, and adaptable across use cases.

### 2.2. Optimization-Based Motion Planning

While sampling-based planners have proven highly effective for exploring complex configuration spaces, they often require post-processing to improve path smoothness and dynamic feasibility. Optimization-based motion planners address this need by formulating the motion planning problem as a continuous optimization problem, where an initial trajectory is iteratively refined to minimize a cost function subject to kinematic and dynamic constraints. These methods are particularly valuable in applications requiring smooth, precise, and dynamically feasible paths, such as robotic assembly, high-speed pick-and-place, and surface processing.

One of the most influential methods in this category is CHOMP [5], which introduced a trajectory optimization algorithm capable of incorporating smoothness and obstacle cost gradients into the optimization process. CHOMP operates in the space of trajectories and uses functional gradient descent to minimize an objective that balances obstacle avoidance with motion smoothness. Although CHOMP requires an initial trajectory to converge, it is highly efficient and has been demonstrated in cluttered environments and constrained spaces.

STOMP [6] builds upon these ideas but introduces a sampling-based update mechanism that does not require gradient information, making it more robust to non-differentiable cost functions. STOMP evaluates multiple noisy perturbations of a trajectory and updates it based on weighted averaging of improved samples. Its robustness and flexibility have made it appealing in scenarios with complex or hard-to-model constraints.

TrajOpt further advances the field by applying sequential convex optimization techniques to trajectory planning problems [7]. TrajOpt linearizes collision constraints and trajectory costs, solving a series of convex subproblems that converge rapidly to locally optimal solutions. This approach offers a good balance between speed and path quality, and has been widely adopted for online trajectory refinement in both simulation and real-time applications.

These optimization-based methods are widely supported by MoveIt, where they are often used as trajectory post-processing modules following an initial plan computed by sampling-based algorithms. They are also well-suited to direct use in constrained industrial tasks where workspace structure or motion policy requirements are well understood, for example, applications involving robotic welding, insertion, or toolpath following, which often benefit from optimization-based planning due to the need for continuity in end-effector motion and precise velocity control.

Despite their advantages, optimization-based planners are inherently local: their performance depends heavily on the quality of the initial trajectory, and they may fail to converge in environments with complex constraints or narrow passages. To mitigate this, they are frequently embedded within hybrid planning pipelines, where a sampling-based planner provides a feasible initialization that is then refined using an optimization-based solver. This division of labor has become a common pattern in industrial and research applications alike, enabling the construction of planners that are both reliable and high-performing.

### 2.3. Hybrid Planning Approaches

In practice, no single motion planning paradigm fully satisfies the diverse requirements of modern robotic systems, particularly in industrial settings that demand both reliability and high-performance motion generation. As a result, hybrid planning architectures have emerged that combine the strengths of sampling-based and optimization-based planners within unified pipelines. These hybrid methods aim to mitigate the limitations of each class of algorithm by exploiting their complementary characteristics.

A common approach in hybrid planning involves using a sampling-based planner such as RRT-Connect [32] or PRM to generate an initial collision-free trajectory, followed by refinement through an optimization-based planner like CHOMP, STOMP, or TrajOpt [33]. This two-stage pipeline allows for global exploration of the configuration space while ensuring the final trajectory is smooth, dynamically feasible, and compliant with end-effector constraints. Several studies have demonstrated the effectiveness of this approach for applications involving obstacle avoidance in tight spaces, precision path tracking, and multi-step industrial tasks.

The integration of OMPL as a pre-processor for optimization-based planners like CHOMP and STOMP is a common practice in standardized motion planning pipelines such as MoveIt. This hybrid approach leverages the strengths of both sampling-based and optimization-based methods to achieve efficient and high-quality motion planning [34,35]. OMPL planners are used to generate an initial collision-free trajectory in the configuration space. This trajectory serves as a seed for CHOMP/STOMP, which then refines it to meet additional constraints such as smoothness, obstacle clearance, and dynamic feasibility. The initial trajectory provided by OMPL ensures that CHOMP/STOMP starts from a feasible solution, significantly reducing the risk of convergence to local minima and improving overall planning efficiency. MoveIt provides a modular framework that seamlessly integrates OMPL and optimization-based planners. This hybrid pipeline combines the global exploration capabilities of OMPL with the local refinement strengths of CHOMP/STOMP, offering improved convergence, higher-quality trajectories, and modularity for diverse applications.

Recent extensions of this idea include dynamic planning architectures, where replanning is performed in real time by alternating between fast, sparse sampling and rapid trajectory refinement steps [36]. Additionally, frameworks such as the MoveIt Task Constructor enable modular construction of hybrid pipelines by chaining sampling, optimization, and constraint satisfaction stages in a task-specific manner, facilitating deployment in manufacturing cells with varying workspace layouts and task demands.

Hybrid planners have also been deployed in manipulation tasks involving dual-arm or humanoid robots, coordinated motion planning, and tool-use operations, where both global coordination and local precision are critical [37]. In such settings, the initial planner may focus on gross motion feasibility—ensuring collision-free paths between goal poses—while the optimizer enforces task-space requirements such as synchronized movement or compliance with tool constraints.

Beyond traditional planning, hybridization also serves as a foundation for integrating learning-based components into classical pipelines [38]. For example, learned models can predict initial trajectories or bias the sampling distribution, while classical planners ensure feasibility and safety. This synergy between data-driven priors and algorithmic guarantees reflects a growing trend toward “learning-augmented” planning, which is especially attractive in industrial contexts where both reliability and adaptability are paramount.

Recent research has begun to merge sampling-based methods with learning components to overcome their respective weaknesses. For example, learned heuristics have been used to bias the sampling distribution in informed RRT* and BIT* [24,25], reducing search time in complex spaces. Other works integrate neural network predictors to generate warm-start trajectories or estimate cost-to-go, which sampling planners then refine for feasibility and optimality [39]. These hybrid frameworks demonstrate that combining data-driven priors with algorithmic guarantees can yield both fast convergence and high-quality paths, pointing toward a promising direction for industrial motion planning.

As the planning demands of industrial robotics continue to grow—especially with the rise of human–robot collaboration, reconfigurable cells, and mobile manipulation—hybrid architectures are poised to become a standard design pattern. They offer a flexible way to balance global planning robustness with local refinement precision, while also providing a natural interface for integrating optimization, sampling, and learning-based methods into cohesive motion generation systems.

### 2.4. Limitations of Classical Planning Approaches

While both sampling-based and optimization-based motion planning methods have demonstrated significant success across academic and industrial applications, each presents limitations that constrain their applicability in dynamic, time-sensitive, or high-precision industrial scenarios. These limitations become particularly critical in contexts that demand planning determinism, low-latency responses, and consistent integration into real-time control pipelines.

Sampling-based planners such as those implemented in OMPL offer strong generality and probabilistic completeness, but at the cost of stochasticity and computational unpredictability. Even under fixed conditions, planners like RRT-Connect or KPIECE can yield variable performance due to their reliance on random sampling and exploration heuristics [22,40]. This non-determinism leads to varying planning times and success rates, making it difficult to guarantee consistent behavior within tight industrial cycle-time constraints. Furthermore, the paths they generate are often jagged or suboptimal, necessitating post-processing such as shortcutting, spline fitting, or velocity profiling via time-parameterization algorithms like Time-Optimal Trajectory Generation (TOTG) [41].

Optimization-based planners, while capable of producing smoother and more dynamically feasible trajectories, suffer from their own limitations. Methods such as CHOMP, STOMP, and TrajOpt rely heavily on the quality of the initial trajectory and may converge to local minima or fail to find solutions in highly constrained or cluttered workspaces [5,7]. Their computational performance is typically sensitive to the number of waypoints, the presence of sharp cost gradients, and the linearity of collision constraints. Moreover, they often require careful parameter tuning—such as cost weights, step sizes, and regularization terms—which complicates deployment in heterogeneous task setups or dynamically reconfigured environments.

In industrial robotics, these shortcomings have practical implications. Non-deterministic planning latency impedes system-level scheduling and validation. Local convergence failures can block entire workflows if alternative solutions are not readily available. The need for extensive tuning or manual debugging undermines reusability and adaptability, especially in flexible automation cells where robots must be repurposed for different products or workspaces on short notice.

Although hybrid pipelines that combine both approaches can mitigate some of these issues, they introduce additional complexity and often still rely on per-task tuning or human intervention to ensure performance. These persistent challenges motivate the exploration of alternative paradigms, such as learning-based planning, which aim to bypass online search altogether through policy amortization and experience-driven generalization.

### 2.5. Deep Reinforcement Learning for Motion Planning

DRL has emerged as a powerful framework for robot motion planning, particularly in tasks requiring generalization, adaptability, and real-time reactivity. By optimizing policies that map high-dimensional observations to actions through trial-and-error interaction with an environment, DRL methods can learn control strategies that are difficult to specify explicitly or solve analytically [42]. This makes them attractive for robotic manipulation tasks in unstructured or partially observable environments, such as warehouse picking, dynamic assembly, and human–robot collaboration [43,44].

DRL planners, once trained, provide inference–time trajectory generation without the need to explicitly solve a planning problem online. This property contrasts with both sampling-based and optimization-based planners, which typically recompute a solution for each planning query. Algorithms such as SAC [18], Proximal Policy Optimization (PPO) [45], and Twin Delayed Deep Deterministic Policy Gradient (TD3) [46] have been successfully applied to continuous robot control [16,17,47], including reaching, grasping, insertion, and trajectory tracking [13,43]. These methods leverage function approximation via deep neural networks and can scale to high-dimensional action spaces, including joint-space control of 6- or 7-DOF manipulators.

Several research efforts have demonstrated the feasibility of DRL-based motion planning for industrial robots. For example, policies have been trained to solve Cartesian reaching tasks using sparse rewards [44], perform path-constrained tool motions, or follow orientation-sensitive end-effector constraints. These studies often employ curriculum learning or reward shaping to guide training, and some inject expert demonstrations from classical planners to bootstrap learning in sparse reward settings [45]. This ability to integrate prior knowledge aligns DRL with hybrid planning frameworks, allowing it to serve as a global planner, an initialization policy, or a local control module [48].

In the context of motion planning, DRL is particularly well-suited to problems where traditional planners struggle—such as those with partially observed dynamics, unknown cost landscapes, or non-convex constraints. Moreover, DRL policies produce deterministic outputs at inference time, making them appealing in applications where latency and repeatability are critical [49]. For instance, recent benchmarks have shown that DRL-based planners can match or outperform classical planners in both planning time and path smoothness once trained, particularly in structured but dynamic environments [50].

Nevertheless, the adoption of DRL in industrial motion planning remains limited by several factors. Chief among these is the challenge of sample efficiency: policies often require millions of environment interactions to converge. This is compounded by the sim-to-real gap, where discrepancies between simulated and physical environments cause trained policies to fail when deployed on real hardware. Solutions such as domain randomization [51], privileged training, and fine-tuning with real-world demonstrations have been proposed to mitigate these challenges, but their deployment in time-constrained manufacturing setups remains an open problem [52].

Recent years have also seen the emergence of standardized, robot-centric DRL benchmarks that include reaching and motion generation tasks for multi-DoF arms. PandaGym provides goal-conditioned reaching and manipulation tasks for the Franka arm with strong baselines for SAC/TD3/PPO and reproducible evaluation protocols [53]. Robosuite offers a modular MuJoCo-based suite with arm-centric reaching/manipulation tasks and controllers, widely used for algorithmic comparisons [54]. RLBench contributes a large set of manipulation tasks (including reaching as a primitive) designed to stress perception and control and is frequently used to test generalization across task variants [55]. Complementing these platforms, recent surveys synthesize progress and open challenges in applying DRL to robotic control and planning, emphasizing lessons for real-world deployment and evaluation [56]. On the application side for manipulators, recent studies continue to deploy SAC/PPO/TD3 variants for collision-aware arm trajectory planning and human-aware avoidance, highlighting typical pain points (reward shaping, sample efficiency, stability) that motivate hybridization with classical modules and stronger benchmarking [57,58].

Alternative intelligent control strategies for robotic arms have also been proposed outside of the DRL paradigm. For instance, adaptive critic designs have been applied to achieve safety-optimal Fault-Tolerant Control (FTC) in nonlinear systems with asymmetric constrained inputs [59], demonstrating how actor–critic architectures can be tailored for safety-critical operation. Similarly, event-triggered $H_{\infty }$ control schemes have been developed for unknown constrained nonlinear systems, with direct applications to robotic arms [60] offering robustness guarantees under limited actuation and dynamic uncertainties. While these approaches differ from the DRL-based motion planning considered in this work, they share the goal of enabling the safe and efficient operation of robotic manipulators under real-world constraints.

In contrast to platform-oriented or algorithm-proposal papers, our contribution is a large-scale, ROS/MoveIt-parity comparison between OMPL sampling-based planners and an SAC-based planner under identical pipeline adapters and post-processing. Methodologically, our DRL training uses a coupled position–orientation curriculum (rather than independent thresholds) and a dual-use dataset (benchmark + expert demonstrations) built from uniform workspace sampling with distance-balanced start–goal pairs. Prior DRL reaching or motion-planning works do not report head-to-head comparisons with OMPL under ROS-equivalent interfaces at this scale nor enforce coupled pose accuracy as an explicit training constraint [53,54,55,56].

### 2.6. Comparative Benchmarks and Research Gaps

Systematic benchmarking plays a crucial role in evaluating and comparing the performance of motion planning algorithms. Over the past decade, several studies have attempted to provide structured evaluations of classical planning strategies, often focusing on sampling-based planners due to their algorithmic diversity and widespread adoption in practical robotics. Frameworks such as the MoveIt benchmarking suite and MotionBenchMaker [61] have enabled quantitative comparisons across planners like RRT, PRM, KPIECE, and BIT*, using metrics such as planning success rate, computation time, and trajectory smoothness. These tools have helped identify strengths and limitations across a range of scenarios, including cluttered environments, narrow passages, and high-DOF manipulators. Other resources such as Planner Arena [62] provide visual analytics and standardized reporting for OMPL benchmarking results, allowing performance to be explored interactively. While these infrastructures have been instrumental for evaluating classical planners, they are primarily designed for scene-centric evaluations and do not directly accommodate the requirements of benchmarking learning-based planners, such as large-scale uniform workspace sampling, redundancy exposure, or replay-buffer data generation.

Kroemer et al. [63] provide an extensive overview of the role of machine learning in robot manipulation. They explore the challenges of building robots that can effectively interact with their environment and manipulate objects to achieve specific goals. The paper emphasizes that traditional approaches often fail to capture the complexity and variability of real-world tasks, making learning-based techniques essential. The authors discuss various machine learning approaches used in robot manipulation, including deep learning and reinforcement learning, and propose a unified framework for understanding these methods. Their work identifies key research opportunities in the field, such as improving the efficiency of learning algorithms and enhancing their ability to generalize across diverse tasks and environments. This research serves as a foundational reference for the integration of machine learning in robotic manipulation, directly influencing the design of advanced motion planning algorithms for industrial applications.

Elbanhawi et al. [12] focus on the computational efficiency and limitations of classical sampling-based motion planning algorithms, such as RRT and PRM. They discuss the probabilistic completeness of these methods, which guarantees a solution if one exists, but also emphasize that the probability of reaching an optimal solution diminishes over time. To address this limitation, they introduce optimal sampling planners, which offer asymptotic optimality as planning time approaches infinity. However, the authors point out the slow convergence of these planners and propose heuristic and post-processing methods to improve their efficiency. Their work underscores the complexity of tuning sampling-based algorithms and the need for improved convergence rates, highlighting a key challenge in classical motion planning that has driven interest in hybrid approaches combining classical and learning-based methods.

Recent work by Orthey et al. [22] provided one of the most comprehensive comparative reviews of sampling-based planning to date, analyzing the empirical performance of over 40 planners on 25 motion planning problems. Their study emphasized that no single planner is universally superior and that performance varies significantly depending on problem structure. These findings reinforce the importance of benchmarking under realistic, application-specific conditions—particularly in industrial contexts where robustness and determinism are key operational requirements.

Despite these advances, the literature on DRL-based motion planning remains fragmented, with few studies providing direct comparisons to classical planners. Most existing benchmarks focus on either simulation environments or specific robotic platforms, making it difficult to generalize findings across different setups. Furthermore, many studies evaluate DRL policies in isolation, without considering their performance relative to established classical methods or to a limited set of planners [15,16,47]. This lack of comparative analysis limits our understanding of the strengths and weaknesses of DRL in practical applications and hinders the development of hybrid approaches that leverage both classical and learning-based techniques.

To address both the lack of DRL–classical parity and the limitations of existing benchmarking tools, our dataset construction departs from scene-centric approaches like MotionBenchMaker and Planner Arena in several key ways. First, we employ uniform spherical sampling of the reachable workspace, followed by distance-binned start–goal pairing to balance problem difficulty across short-, medium-, and long-range motions. Second, we use IKFast to enumerate all possible inverse-kinematics solutions for each pose, filtering out self-collisions and floor collisions to retain only feasible configurations while exposing the robot’s kinematic redundancy. Third, we ensure strict ROS/MoveIt parity by running both OMPL planners and the DRL planner through identical request adapters and time-parameterization stages (TOTG/TOPPRA), guaranteeing fairness in execution and timing. Finally, our dataset serves a dual purpose: it supports both large-scale benchmarking of classical planners and provides high-quality expert demonstrations for seeding DRL replay buffers, a feature not supported by existing benchmarking frameworks.

Our work aims to address this gap by constructing a shared benchmark that compares OMPL-based sampling planners and a SAC policy trained with expert demonstrations across over 100,000 motion queries for an industrial robot arm. By standardizing the dataset, evaluation metrics, and planning interfaces, we provide an empirical foundation for assessing how classical and learning-based motion planning approaches perform under operationally relevant conditions.

## 3. Materials and Methods

This section describes the experimental methodology employed to compare classical sampling-based motion planning techniques with DRL approaches for trajectory generation on a UR3e robotic manipulator. The experimentation was structured in two main phases. First, a large-scale dataset of over 100,000 motion planning trajectories was generated by systematically sampling the robot’s reachable workspace and solving motion queries using OMPL-based planners within the MoveIt framework. These trajectories served both as a benchmark for classical methods and as expert demonstrations to accelerate DRL training. In the second phase, SAC policy was trained in simulation to reproduce the motion planning task, using the dataset to bootstrap learning and improve sample efficiency. Both the OMPL-generated and DRL-generated trajectories were post-processed using time-optimal parameterization algorithms to ensure dynamic feasibility, enabling a fair comparison across methods in terms of planning success rate, trajectory quality, and execution consistency.

### 3.1. Dataset Generation

A dataset of over 100,000 trajectories was generated to evaluate and compare the performance of traditional motion planning techniques and DRL for motion planning in the UR3e robotic manipulator equipped with an OnRobot RG2 gripper. The dataset was constructed in two phases:

1. Uniform sampling of the workspace and filtering of feasible configurations;
2. Generation of full trajectories using all the available planners in MoveIt’s OMPL planning pipeline, ensuring only valid and feasible motions were included.

#### 3.1.1. Workspace Definition and Uniform Pose Sampling

The workspace W of the UR3e robot is defined as the set of all possible end-effector Cartesian positions and orientations that can be reached while maintaining a valid joint configuration. Mathematically, we express this as:

$$W=\left\{\left(x,y,z,R\right)\in R^{3}\times SO\left(3\right)\;|\;\exists q\in C,\;f\left(q\right)=\left(x,y,z,R\right)\right\}$$

where

- (x,y,z) denotes the Cartesian position of the end-effector.
- R∈SO(3) represents its orientation.
- q∈C is a valid joint configuration.
- f:C→W is the forward kinematics function.

To ensure that the workspace was sampled in a representative manner, a spherical uniform distribution was used to generate feasible end-effector positions. The poses were sampled within a bounded workspace range to avoid collisions with the base and maximize coverage of reachable areas. Furthermore, for each pose, multiple orientation values were sampled to ensure a diverse representation of feasible end-effector configurations. The sampling followed a hemispherical constraint, ensuring no samples were generated in unreachable regions directly below the base. The limits were defined as follows:

- Radial distance: r∈[0.1,0.85] meters to prevent self-collisions near the base and ensure reachability within the robot’s range.
- Azimuthal angle θ: Uniformly sampled in [0,2π], covering all directions in a circular plane.
- Elevation angle ϕ: Sampled using ϕ=arccos(1−u) (where u∼U(0,1)), ensuring uniform distribution in the hemisphere.
- Orientation: Random joint configurations were generated for each sample, within the UR3e’s joint limits.

The mathematical formulation of the sampling is as follows:

$$\begin{array}{cc} r & =U(r_{\min},r_{\max})\\ \theta & =U(0,2\pi )\\ \phi & =\arccos(1-U(0,1)) \end{array}$$

where U(a,b) denotes a uniform distribution between *a* and *b*. The sampled Cartesian coordinates of the poses are then computed as:

$$\begin{array}{cc} x & =r\cos\theta \sin\phi \\ y & =r\sin\theta \sin\phi \\ z & =r\cos\phi \end{array}$$

To ensure comprehensive workspace coverage, an iterative process was conducted to determine the optimal number of sampled poses. Initially, multiple pose number sizes were tested by varying the number of uniformly sampled poses. Each variation was graphically analyzed to verify that all regions of the workspace were adequately represented without excessive clustering or gaps. This process was repeated, progressively refining the number of poses, until a balance was achieved as follows:

- The entire reachable workspace was covered.
- No regions were overrepresented.
- The sampling maintained uniformity in the distribution of poses.

**Figure 1:**
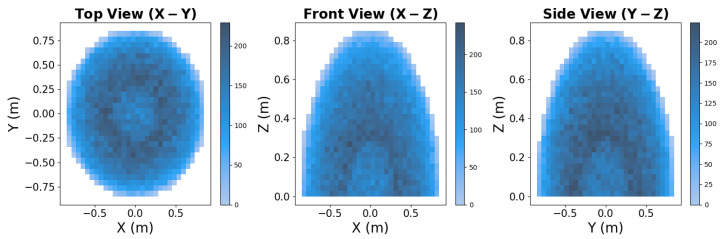
Workspace distribution of uniformly sampled goal positions for the robot. The figure presents three different views (Top, Front, and Side) to illustrate the uniform coverage of the reachable space.

Figure 1 illustrates the final validated distribution of poses, showing uniform coverage of the reachable workspace. Notably, an inner spherical region remains unoccupied due to proximity to the base, preventing self-collisions. A total of 134,742 poses were sampled, ensuring a diverse representation of the robot’s workspace.

#### 3.1.2. Inverse Kinematics Computation and Configuration Space Filtering

**Figure 2:**
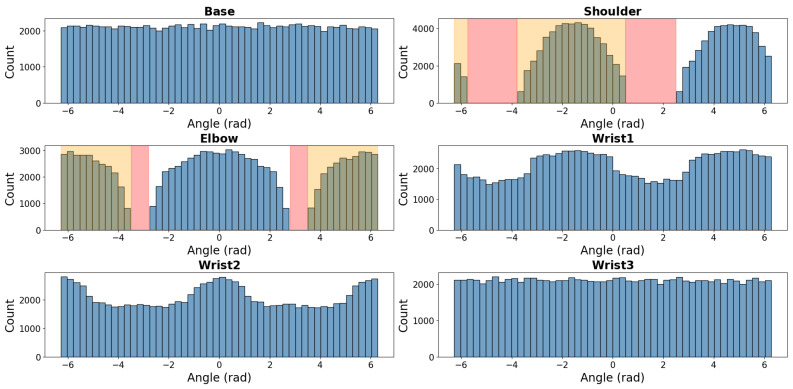
Joint configuration distribution for the initially sampled poses for the UR3e robot before filtering. The sampled space contains unreachable regions (orange) and collision constraints (red).

For each sampled workspace pose, all possible Inverse Kinematic (IK) solutions were computed using IKFast [64], an analytical solver optimized for efficiency. Given a workspace pose (x,y,z,R), the corresponding set of joint-space solutions was defined as:

$$Q\left(x,y,z,R\right)=\{q_{i}\in C|f\left(q_{i}\right)=\left(x,y,z,R\right)\}$$

where Q represents all possible joint configurations satisfying the given pose. However, not all computed configurations were viable for execution, as some may lead to infeasible transitions between configurations or kinematic constraints. To ensure that the final set of sampled poses and configurations allows for valid motion planning, the following filtering steps were applied:

1. **Self-collision filtering**: Configurations that resulted in self-collisions between any part of the robot’s links were discarded.
2. **Floor collision filtering**: In configurations where the end-effector or other parts of the robot intersected, the floor was removed.
3. **Unreachable configurations**: If no valid IK solutions existed for a given pose or the configuration was not reachable due to physical constraints, the pose was discarded (see Figure 2).

The purpose of this filtering was to ensure that for any chosen pose’s initial configuration, it is kinematically possible to transition to a valid goal pose’s configuration through a feasible trajectory. By removing configurations that are not physically achievable or that create isolated solutions in the configuration space, this step guarantees that motion planning algorithms can consistently generate collision-free and executable trajectories.

Figure 2 shows the initial configuration space distribution for all the sampled poses before filtering, where many sampled pose configurations were found to be infeasible due to self-collisions, floor collisions, or unreachable workspace regions. The first pose sampling phase provided a large set of candidate configurations, but a significant portion of them were non-executable due to physical and kinematic limitations of the robot.

To extract a refined set of valid poses and associated joint configurations, a filtering algorithm was applied. This algorithm systematically iterated through the initially sampled configurations, discarding those that violated reachability, self-collision, or floor constraints. The details of this process are outlined in Algorithm 1.
**Algorithm 1** Filtering of Valid Poses and Associated Joint Configurations1:**Input:** Robot kinematic model, workspace bounds W2:**Output:** Set of valid poses and associated configurations C={(x,y,z,R,q)}3:Uniformly sample over 100,000 poses in the robot’s workspace4:Initialize set of valid configurations C←∅5:**for** each sampled pose (x,y,z,R)∈W **do**6:   Compute all IK solutions Q(x,y,z,R) using IKFast7:   **for** each joint configuration $q_{i}\in Q$ **do**8:       **if** $q_{i}$ is collision-free and reachable **then**9:          Add $\left(x,y,z,R,q_{i}\right)$ to *C*10:     **end if**11:   **end for**12:**end for**13:**Return** *C*

**Figure 3:**
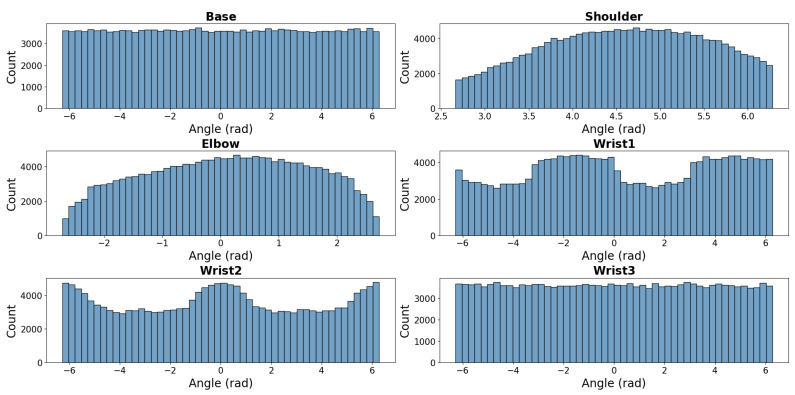
Configuration space sample distribution after filtering unreachable regions and collision constraints.

After applying the filtering constraints, 104,295 valid initial and goal poses along with their corresponding joint configurations were selected. The final configuration space distribution, displayed in Figure 3, presents a refined set of poses containing only valid and executable joint configurations. This selection ensured the following:

- Diversity of trajectory complexity, including short, medium, and long-distance movements.
- Even coverage of the workspace, ensuring that all regions of W were represented.
- Balanced representation of joint configurations, preserving uniformity in the valid configuration space.

#### 3.1.3. OMPL-Based Motion Planning Pipeline

This subsection describes the internal motion planning pipeline used by MoveIt when interfacing with the OMPL for sampling-based global motion planning. The pipeline transforms a high-level pose goal into a fully time-parameterized, dynamically feasible trajectory in configuration space through several modular stages.

The process begins with the user-defined input: a target pose in Cartesian space, specified for the robot’s end-effector. Since OMPL operates in configuration space, this pose goal must first be converted into one or more joint-space configurations. This is accomplished through the kinematics plugin, in this case, IKFast, which provides fast and analytical IK solutions. A valid goal configuration is selected among those returned by the plugin, taking into account feasibility constraints in the configuration space.

Following this conversion, the motion planning request is internally constructed and processed by a series of pre-processing planning request adapters. These adapters serve to prepare and sanitize the request before it reaches the planner. Common adapters used at this stage include the following:

- CheckStartStateBounds: Ensures that the start state is within joint limits.
- ValidateWorkspaceBounds: Confirms that the start and goal are within the declared workspace.
- CheckStartStateCollision: Verifies that the initial configuration is collision-free.
- ResolveConstraintFrames: Resolves any relative frames used in the constraint specification.

After pre-processing, the validated motion planning request is forwarded to the OMPL motion planner. At this stage, OMPL attempts to solve the motion planning problem in configuration space using the requested planner (e.g., RRT, RRTConnect, PRM, among others). The planner incrementally builds a roadmap or tree by sampling random configurations and connecting them while enforcing kinematic limits, collision avoidance (using FCL), and workspace constraints. The result of this step is a raw path, i.e., a sequence of joint-space waypoints connecting the start and goal configurations.

Once a valid path is found, MoveIt begins constructing the motion planning response. This response includes the raw trajectory, as well as status information, about the planning attempt. Before finalizing the response, a second sequence of adapters—known as post-processing request adapters—is applied to refine the trajectory further. Typical post-processing steps include the following:

- AddTimeOptimalParameterization: Applies time-optimal path parameterization (TOTG) [41] to assign velocity and timing profiles consistent with the robot’s dynamic constraints.
- ValidateSolution: Ensures that the final trajectory remains collision-free and satisfies all dynamic and kinematic constraints.

The final output is a fully parameterized, smooth, and executable trajectory in configuration space. This trajectory can be passed directly to the controller for execution or used offline for training and benchmarking, as in the case of this study.

#### 3.1.4. Trajectory Dataset Construction

**Figure 4:**
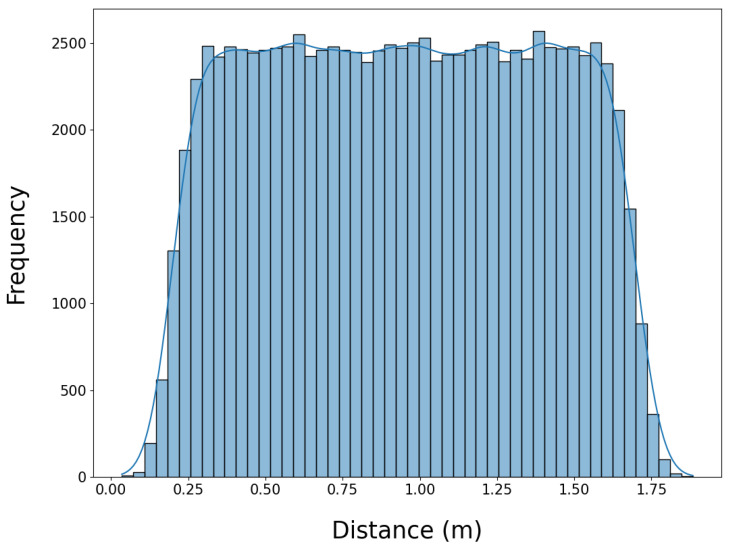
Distribution of sampled start and goal pose distances in the workspace.

The dataset construction began with the set of valid poses and their associated joint configurations obtained from the configuration space filtering process. To ensure a uniform distribution of motion trajectories across different workspace path lengths, distance bins were defined, allowing pose pairs to be sampled evenly based on their separation in the workspace. Figure 4 illustrates the final distribution of sampled start and goal pose distances in the workspace. The distance bins were designed to ensure that the dataset contained a balanced representation of short, medium, and long-distance movements, providing a diverse set of motion planning scenarios.

For each sampled start and goal pose pair, a random valid configuration was selected for the initial pose, and planning requests for every available OMPL planner using MoveIt’s motion planning pipeline were made. To ensure a correct trajectory generation, this pipeline was configured as follows:

- The IKFast kinematics plugin was used to compute the IK for the goal pose. The plugin was configured to use the filtered constraints identified during the configuration space filtering.
- OMPL planners were configured to attempt path planning with a maximum of 10 attempts per trajectory and a 5 s timeout per attempt.
- The time-optimized trajectory was obtained using MoveIt’s TOTG module, with a 0.1 scaling factor for the velocity and acceleration limits.

For every pose pair, the pipeline was executed for all available planners in MoveIt’s OMPL motion planning pipeline. Algorithm 2 outlines the overall process for constructing the trajectory dataset. The successful trajectories were recorded, along with relevant data consisting of full motion trajectories, not just pose pairs. For each successfully planned trajectory, the following data was recorded:

- Planner name: Identifying which OMPL planner successfully computed the trajectory.
- Initial pose: The start pose of the motion plan.
- Goal pose: The target pose of the motion plan.
- Path: The sequence of joint configurations representing the motion plan.
- Time parameterization: The time-optimized trajectory obtained using TOTG [41].
- Planning time: The total computation time required to generate the trajectory.
- Configuration space length: The total length of the path in the configuration space.

The initial and final configurations were stored along with the path data to ensure reproducibility and consistency in the dataset.
**Algorithm 2** Trajectory Dataset Construction1:**Input:** Set of valid poses and associated configurations *C*2:**Output:** Final dataset $D=\{\left(P_{\mathrm{start}},P_{\mathrm{goal}},\tau ,\mathrm{planner},\mathrm{param},\mathrm{time},\mathrm{length}\right)\}$3:Initialize dataset D←∅4:Define distance bins for sampling pose pairs5:**for** each distance bin *d* **do**6:   Uniformly sample pose pairs $\left(p_{\mathrm{start}},p_{\mathrm{goal}}\right)$ from *C* within distance *d*7:   **for** each $\left(p_{\mathrm{start}},p_{\mathrm{goal}}\right)$ **do**8:     Randomly select a valid configuration $q_{\mathrm{start}}$ from $Q(p_{\mathrm{start}})$9:     Compute IK for $p_{\mathrm{goal}}$ using IKFast solver with filtered constraints10:     **for** each OMPL planner *P* in MoveIt **do**11:        **for** attempt i=1 to 10 **do**12:          Attempt trajectory planning $\tau =P(q_{\mathrm{start}},q_{\mathrm{goal}})$ with a 5-second timeout13:          **if** τ is valid **then**14:             t = TOTG(τ)15:             Compute configuration space length of τ16:             Store (*P*, $P_{\mathrm{start}}$, $P_{\mathrm{goal}}$, τ, t, planningtime, configlength) in *D*17:          **end if**18:        **end for**19:     **end for**20:   **end for**21:**end for**22:**Return** *D*

The planning process was conducted for all 104,295 pose pairs, obtaining the same number of trajectories. The pose and configuration pair distribution ensured that the dataset contained a balanced representation of short, medium, and long-distance movements, providing a diverse set of motion planning scenarios.

The dataset was divided into training, validation, and test sets, with 80%, 10%, and 10% of the trajectories, respectively. The training set was used to train the DRL agent, while the validation and test sets were used to evaluate the performance of both classical and DRL-based motion planning methods.

### 3.2. Deep Reinforcement Learning Formulation and Training

This section describes how the motion planning task was formulated as a Markov Decision Process (MDP) and solved using DRL. The agent was trained in a simulated environment using a hybrid reward function, expert demonstrations, and curriculum learning [65] to progressively acquire high-precision motion skills across the robot’s full workspace.

#### 3.2.1. Problem Formulation

The motion planning task was modeled as a finite-horizon, episodic MDP defined by the tuple (S,A,P,r,γ), where S denotes the state space, A the action space, *P* the transition dynamics, *r* the reward function, and γ the discount factor.

The agent observes the robot’s current joint states, the relative pose to the goal, and a collision indicator. It outputs incremental joint motions as actions. The environment transitions deterministically based on the commanded joint updates, with collisions and kinematic constraints modeled through PyBullet [66].

#### 3.2.2. Environment Design and State–Action Spaces

To train the reinforcement learning agent for motion planning, a custom environment was implemented using the PyBullet physics engine. The simulated setup included a UR3e robotic manipulator equipped with an OnRobot RG2 gripper and a planar floor object restraining the robot’s workspace. The UR3e robot was modeled using the URDF format, and the gripper was attached to the end-effector link. The simulation environment was designed to closely resemble the real-world setup, including the robot’s kinematic and dynamic properties, as well as collision detection capabilities. It was configured to model only the kinematics and collision checking of the robot. Dynamics were omitted to speed up the training process, as the trajectory generation problem focuses solely on geometric feasibility and does not require consideration of dynamic constraints. The environment also included a planar floor object to enable detection of potential floor collisions.

The reinforcement learning agent interacted with the environment through a structured observation and action interface. The observation space O was composed of 14 continuous floating-point values, ensuring that the policy has all the necessary information to generalize across the entire robot workspace. This is particularly important because if only the poses were provided, the non-unique mapping between the workspace and configuration space would prevent the policy from generalizing effectively. The observation space includes the following:

- The six current joint values of the robot, $q=\left[q_{1},\ldots ,q_{6}\right]\in R^{6}$
- The Cartesian difference between the end-effector’s current pose and the goal pose:
  $$\Delta p=p_{\mathrm{goal}}-p_{\mathrm{current}}\in R^{3},$$
  $$\;\Delta r=\mathrm{quatdiff}\left(r_{\mathrm{goal}},r_{\mathrm{current}}\right)\in R^{4}$$
  where the quaternion difference quatdiff is computed as:
  $$\mathrm{quatdiff}\left(r_{\mathrm{goal}},r_{\mathrm{current}}\right)=r_{\mathrm{goal}}⊗r_{\mathrm{current}}^{-1}$$
  Here, ⊗ denotes the quaternion multiplication operator, and $r_{\mathrm{current}}^{-1}$ is the inverse of the current quaternion, defined as:
  $$r_{\mathrm{current}}^{-1}=\left[r_{w},-r_{x},-r_{y},-r_{z}\right]$$
  for a quaternion $r_{\mathrm{current}}=\left[r_{w},r_{x},r_{y},r_{z}\right]$.
- A binary collision indicator:
  $$c=\left\{\begin{array}{cc} 1.0 & \mathrm{if}\;\mathrm{in}\;\mathrm{self}\;\mathrm{or}\;\mathrm{floor}\;\mathrm{collision}\\ 0.0 & \mathrm{otherwise} \end{array}\right.$$

Therefore, the full observation vector is:

$$o=\left[q_{1},\ldots ,q_{6},\Delta x,\Delta y,\Delta z,\Delta o_{w},\Delta o_{x},\Delta o_{y},\Delta o_{z},c\right]\in R^{14}$$

The action space A was defined as a 6-dimensional continuous vector representing joint increments in radians, constrained within a fixed range for each joint:

$$a=\Delta q=\left[\Delta q_{1},\ldots ,\Delta q_{6}\right]\in {\left[-0.1,0.1\right]}^{6}$$

Here, the action space forms a continuous box, where each joint increment $\Delta q_{i}$ can vary independently within the interval [−0.1,0.1]. These increments are applied additively to the current joint state at every step and are clipped to ensure they remain within the physical joint limits of the UR3e robot.

The problem exhibits a high-dimensional and continuous state space, involving six joint angles and seven pose deltas (position and orientation as quaternions), plus a discrete collision state. This yields a 14-dimensional hybrid observation vector with both continuous and categorical components. Furthermore, the state space is highly non-linear due to the forward kinematics and collision constraints imposed by the robot geometry. These properties significantly increase the complexity of the exploration and learning process, making standard RL strategies difficult to converge. As such, careful design of the reward function and additional training strategies (such as expert demonstrations and curriculum learning) were necessary for facilitating effective policy optimization.

#### 3.2.3. Curriculum Learning

To facilitate progressive learning and stabilize convergence in this high-dimensional motion planning task, a dual-curriculum learning strategy was implemented. The curriculum controlled the difficulty of the task by gradually tightening the tolerances required for successful completion:

- Position precision $\varepsilon_{p}$: distance between the current and goal Cartesian positions in meters.
- Orientation precision $\varepsilon_{r}$: angular distance between the current and goal orientations (in quaternion form).

Progression to the next curriculum level was triggered after the agent completed a fixed number of consecutive successful episodes (typically 100, to ensure proper progress and reach the expected tolerances) under the current tolerances. This mechanism allowed the policy to gradually master coarse-to-fine motion precision while maintaining high exploration and learning signals.

Both position and orientation curricula were coupled, requiring the agent to improve simultaneously in translational and rotational dimensions. This approach ensured balanced learning for the UR3e robot, addressing its rotational redundancy and joint constraints while maintaining consistent progress across both position and orientation accuracy. This coupling also prevented the agent from early focusing on reaching high accuracy in one dimension while neglecting the other, which could lead to suboptimal behaviors or local minima.

#### 3.2.4. Reward Function

Several reward strategies were initially tested, including sparse and dense formulations. However, neither proved sufficient on its own to ensure stable training convergence or generalization.

Sparse rewards, which provided a fixed positive reward only when the agent reached the goal, led to sparse credit assignment and poor exploration [67]. On the other hand, dense rewards based on step-wise penalties proportional to Cartesian or configuration space distances resulted in premature convergence to suboptimal behaviors, such as local oscillations near the goal [68].

As a result, a hybrid reward strategy was adopted. The final reward function combined a dense negative step reward with a sparse success reward. At each timestep *t*, the reward was calculated as:

$$r_{t}=-\alpha \cdot d_{p}\left(t\right)-\beta \cdot d_{r}\left(t\right)+\varphi_{\mathrm{goal}\;\mathrm{reached}}\cdot r_{\mathrm{success}}^{\left(i\right)}$$

where:

- $d_{p}\left(t\right)$ is the Euclidean distance between the end-effector position and the target position in meters, computed as:
  $$d_{p}\left(t\right)={∥p_{t}-p_{\mathrm{goal}}∥}_{2}$$
- $d_{r}\left(t\right)$ is the orientation error, computed as the quaternion angular distance (rad), computed as:
  $$d_{r}\left(t\right)=\arccos\left(2{\langle r_{t},r_{\mathrm{goal}}\rangle }^{2}-1\right)$$
- α and β are scalar weights for position and orientation penalties.
- $\varphi_{\mathrm{goal}\;\mathrm{reached}}$ is an indicator function that activates when both $d_{p}\left(t\right)<\varepsilon_{p}^{\left(i\right)}$ and $d_{r}\left(t\right)<\varepsilon_{r}^{\left(i\right)}$.
- $r_{\mathrm{success}}^{\left(i\right)}$ is the success reward, which scales with the current curriculum level *i*.

The curriculum defines progressively stricter thresholds for success:

$$\varepsilon_{p}^{\left(i+1\right)}=\max\left(\varepsilon_{p}^{\left(i\right)}\cdot \delta ,\varepsilon_{p}^{\mathrm{target}}\right),\;\varepsilon_{r}^{\left(i+1\right)}=\max\left(\varepsilon_{r}^{\left(i\right)}\cdot \delta ,\varepsilon_{r}^{\mathrm{target}}\right)$$

where δ<1 is the decay factor, and $\varepsilon^{\mathrm{target}}$ is the minimum tolerance. The success reward was also scaled inversely with the curriculum level, using:

$$r_{\mathrm{success}}^{\left(i\right)}=\frac{r_{0}}{\delta^{i/4}}$$

This encourages the agent to continue improving even as the task becomes more difficult, maintaining a strong learning signal. Multiple experiments were conducted to determine the optimal values for α, β, success reward $r_{\mathrm{success}}^{\left(i\right)}$, the curriculum levels, and decay factor δ. The final values were selected based on empirical performance and convergence speed. The final values used in the training process were as follows:

- α=0.2;
- β=10;
- $r_{0}=300$;
- Curriculum levels: 100;
- Decay factor δ=0.98;
- Success thresholds: $\varepsilon_{p}^{\mathrm{target}}=0.0005\;m$, $\varepsilon_{r}^{\mathrm{target}}=0.1\;\mathrm{rad}$;
- Maximum number of consecutive successful episodes to trigger curriculum progression: 100;
- Maximum number of steps per episode: 200.

At the start of training, the tolerances were set to relaxed values $\varepsilon_{p}^{\left(0\right)}=0.5\;m$ and $\varepsilon_{r}^{\left(0\right)}=0.8\;\mathrm{rad}$, making it easier for the agent to succeed.

This dual curriculum strategy ensured that the agent first learned coarse reaching behaviors and then progressively refined its precision, ultimately enabling it to solve the full task under tight tolerances.

At curriculum level *i*, the success predicate requires simultaneous satisfaction of position and orientation tolerances:

(1) $$S^{\left(i\right)}\;=\;\left\{\left(d_{p},d_{r}\right)\;|\;d_{p}<\varepsilon_{p}^{\left(i\right)}\;\wedge \;d_{r}<\varepsilon_{r}^{\left(i\right)}\right\}$$

Advancement from level *i* to i+1 is triggered only after $N_{\mathrm{succ}}=100$ consecutive successful episodes under $S^{\left(i\right)}$. The sparse success bonus $r_{\mathrm{success}}^{\left(i\right)}$ is granted exclusively when $\left(d_{p},d_{r}\right)\in S^{\left(i\right)}$; partial satisfaction (only one metric within tolerance) yields no bonus and never triggers level advancement. This coupling prevents the policy from optimizing one objective at the expense of the other.

Although a 6-DoF manipulator tracks a 6-DoF end-effector pose, it exhibits discrete IK redundancy. For each sampled pose, we enumerate all IKFast solutions Q(x,y,z,R) and retain collision-free ones (Section 3.1.2). Decoupled curricula allow policies to minimize position error while letting orientation drift, often leading to late-stage wrist flips or branch changes that increase path length and risk joint-limit proximity. By contrast, the coupled predicate $S^{\left(i\right)}$ regularizes training toward trajectories that respect both objectives throughout the episode, reducing branch changes and orientation drift.

### 3.3. Coupled vs. Decoupled Pose Curricula

To validate the design choice of coupling position and orientation tolerances during curriculum learning, we compared the proposed coupled strategy against a decoupled variant in which position and orientation thresholds were tightened independently. Both agents used identical SAC architectures, hyperparameters, and training conditions. A 2,000,000 step training budget was allocated to each agent, with performance evaluated on a held-out test set.

The coupled curriculum produced trajectories that maintained orientation accuracy throughout the motion, avoiding late-stage wrist flips and abrupt joint changes observed in the decoupled case. This led to paths that were generally shorter, smoother, and more consistent, with fewer configuration switches between Inverse Kinematic (IK) branches. In contrast, the decoupled variant often achieved good positional accuracy early, but allowed significant orientation drift that required corrective maneuvers near the goal, resulting in longer and less stable motions.

These observations align with the discrete redundancy characteristics of a 6 DoF manipulator: by enforcing simultaneous satisfaction of position and orientation objectives during training, the coupled approach regularizes the policy toward solutions that respect both constraints throughout the motion, improving execution stability without the need for post-hoc corrections.

#### 3.3.1. Episode Structure

Each training episode was initialized by randomly selecting an initial and goal pose from the previously generated dataset of valid configurations. One valid joint configuration was chosen for each pose using the associated IK solutions. The robot was reset to the initial configuration, and the goal pose was provided as part of the observation vector. The episode proceeded with the agent applying incremental joint actions at each step until one of the following termination conditions was met:

1. The end-effector reached the goal within the current curriculum tolerances; this is the success condition defined as follows:
  $$d_{p}\left(t\right)<\varepsilon_{p}^{\left(i\right)}\;\mathrm{and}\;d_{r}\left(t\right)<\varepsilon_{r}^{\left(i\right)}$$
2. A collision with either the robot itself or the floor occurred;
3. A maximum number of steps per episode was reached (typically 200, ensuring that the agent had sufficient time to reach any goal and to explore the environment).

This episode structure ensured diverse trajectory samples while enforcing physical feasibility and encouraging the agent to learn a policy that can generalize across the entire workspace.

#### 3.3.2. Training Algorithm and Hyperparameter Optimization

The training process was implemented using the Stable-Baselines3 (SB3) [69] framework, which provides a robust and modular implementation of state-of-the-art reinforcement learning algorithms. SB3 was chosen for its widespread adoption in the research community, the maturity of its implementation, the extensive validation of its reinforcement learning algorithms, and ease of integration with custom Gymnasium environments [70].

Due to the high dimensionality and non-linear constraints of the motion planning problem, selecting an appropriate reinforcement learning algorithm was critical for achieving stable and efficient learning. An iterative evaluation process was carried out, comparing several state-of-the-art off-policy and on-policy algorithms, including PPO, TD3, and SAC.

Among these, SAC demonstrated the most promising results in terms of convergence speed, stability, and final performance [56]. SAC is particularly well-suited for this task due to its entropy-regularized objective, which promotes exploration in large and continuous action spaces. Furthermore, its off-policy nature allows more efficient reuse of past experiences, which is especially beneficial given the size and complexity of the state–action space involved in robotic motion planning.

The selected actor and critic networks were implemented as Multi-Layer Perceptrons (MLPs) with two hidden layers, each containing 256 units and ReLU activation functions. The output layer of the actor network was configured to produce continuous actions, while the critic network was designed to output Q-values for the state–action pairs. The policy was trained using the soft Bellman backup operator, which incorporates both the Q-value and entropy terms to encourage exploration.

To optimize performance, the hyperparameters of SAC were tuned using a combination of the Optuna framework [71] and empirical results. Optuna, an automatic hyperparameter optimization library based on sequential model-based optimization, was employed to explore the search space for parameters such as learning rates, batch size, entropy coefficient, and target smoothing coefficient. Additionally, empirical testing was conducted to refine the selected hyperparameters further, ensuring robustness and optimal performance across multiple seeds.

The hyperparameter search was conducted over a range of values, including learning rates in $\left[1\times 10^{-5},1\times 10^{-3}\right]$, batch sizes in {1024,2048,4096}, and entropy coefficients in [0.01,0.2]. Each trial was evaluated over 500k environment steps using the validation set success rate as the objective. Our actor and critic networks use two hidden layers of 256 units each with ReLU activations, matching common practice in continuous-control benchmarks and providing sufficient representational capacity without overfitting [18,43].

Optimal values for α, β, success reward $r_{\mathrm{success}}^{\left(i\right)}$ and decay factor δ were also tuned using empirical testing and Optuna. The final values were selected based on a combination of Optuna search results, empirical performance, and convergence speed. The values used in the training process are then presented in the previous section.

The final hyperparameter configuration used for training is summarized in Table 1; they were selected for their balance of convergence speed and stability. Training was performed using mini-batch updates sampled from a replay buffer, with target networks for the value function and policy updated using Polyak averaging.

#### 3.3.3. Expert Demonstrations and Replay Buffer Injection

Despite the careful design of the environment, reward function, and curriculum, training the SAC agent from scratch remained computationally expensive due to the high complexity and dimensionality of the state space. The exploration phase, in particular, suffered from inefficiency during early stages, where the agent often failed to encounter successful trajectories within reasonable timeframes. To address this challenge, a strategy based on expert demonstrations was introduced to accelerate training and improve learning stability.

A set of expert trajectories was readily available from the OMPL-based motion planning dataset described in the previous sections, of which 80% (from the total 104,295 samples) was used for training. Each trajectory was composed of a sequence of collision-free joint configurations connecting a start and a goal pose. To leverage this data, we reconstructed episodes from these trajectories and injected them directly into the SAC agent’s replay buffer.

Each expert trajectory was transformed into a sequence of transition tuples $\left(s_{t},a_{t},r_{t},s_{t+1}\right)$:

- $s_{t}$ was the observation corresponding to the configuration at time step *t*;
- $a_{t}$ was computed as the joint difference $\Delta q=q_{t+1}-q_{t}$;
- $r_{t}$ was computed using the same hybrid reward function as in online training;
- $s_{t+1}$ was the observation after applying $a_{t}$.

The injection strategy followed an adaptive conditional rule: after each training episode, the environment evaluated whether the policy successfully reached the goal within the prescribed tolerances. If the episode failed, a complete expert trajectory was sampled from the dataset and injected in its entirety into the replay buffer. This ensured that the agent could observe full sequences of state–action–reward transitions, leading from an initial state to a successful goal state, rather than isolated transitions.

Once injected, expert and online trajectories coexisted in the buffer and were sampled uniformly during training. Because injections only occurred on failures, the proportion of expert data in the buffer was higher during early training (when failures were common) and decreased naturally as the policy improved. This adaptive mechanism allowed the agent to benefit from high-quality demonstrations when most needed, while avoiding excessive reliance on them later, thus maintaining a healthy balance between exploiting expert knowledge and exploring novel strategies.

#### 3.3.4. Trajectory Post-Processing with TOPPRA

The configuration space paths generated by the trained DRL policy were post-processed using the Reachability-Analysis-based Time-Optimal Path Parameterization (TOPPRA) [72] algorithm, allowing for the production of complete time-parameterized trajectories that respect the robot’s velocity and acceleration limits. TOPPRA is a well-established algorithm for time-optimal trajectory generation, which computes the minimum time required to traverse a given path while satisfying dynamic constraints. The algorithm was applied to the DRL-generated paths to ensure that they were not only kinematically feasible but also dynamically optimal for execution on the UR3e robot. This step enforces dynamic feasibility by respecting joint velocity and acceleration limits.

Each DRL path, composed of a sequence of joint-space waypoints $\left\{q_{0},\ldots ,q_{N}\right\}$, was first interpolated using a quintic clamped spline q(s), where s∈[0,1] is the normalized path parameter. TOPPRA then solved a reachability-constrained optimization problem to compute a time-parameterized trajectory q(t), subject to:

$$\dot{q}\left(s\right)\in \left[-\alpha_{v}\cdot v_{\max},\;\alpha_{v}\cdot v_{\max}\right],\;\ddot{q}\left(s\right)\in \left[-\alpha_{a}\cdot a_{\max},\;\alpha_{a}\cdot a_{\max}\right]$$

with

- $v_{\max}=\left[2\pi ,\ldots ,2\pi \right]\;\mathrm{rad}/s$: conservative joint velocity limits for UR3e.
- $a_{\max}=\left[2\pi ,\ldots ,2\pi \right]\;{\mathrm{rad}/s}^{2}$: conservative joint acceleration limits.
- $\alpha_{v}=0.1$, $\alpha_{a}=0.1$: scaling factors to reflect slower, safer motion profiles.

The time parameterization was computed using TOPPRA’s Seidel solver over a grid of 1000 waypoints. Each trajectory was processed individually, and the time required for parameterization was recorded and added to the original planning time. This enabled a consistent performance evaluation during benchmarking.

#### 3.3.5. Training Process and Hardware Setup

The training process was structured into two distinct phases: an initial phase leveraging expert demonstrations to bootstrap learning, and a subsequent phase, where the agent refined its policy through self-guided exploration and reinforcement learning.

In the initial phase, expert trajectories were incorporated into the agent’s experience replay buffer for every failed episode, providing high-quality samples to accelerate learning and improve early-stage performance. This phase allowed the agent to quickly grasp fundamental motion planning behaviors by imitating expert solutions.

The final phase emphasized self-exploration, enabling the agent to autonomously interact with the environment and refine its policy based on its own experiences. This phase was critical for the agent to generalize beyond the expert demonstrations and adapt to a wide range of scenarios.

Throughout the training process, key performance metrics such as success rate, average reward, and episode length were continuously monitored. Periodic evaluations were conducted to ensure the agent was effectively learning and progressing towards a robust and reliable policy.

The entire training process spanned 6 million environment steps. The hardware setup details are shown in Table 2.

## 4. Results

This section presents the experimental results obtained from the large-scale benchmarking of the DRL planner against classical sampling-based planners from MoveIt’s OMPL planning pipeline. First, we analyze the training performance of the DRL agent, evaluating its learning progression through success rates, reward evolution, curriculum advancement, and episode length over 6 million environment interactions. Subsequently, we compare the DRL-based planner to traditional OMPL planners across the common dataset of motion queries.

### 4.1. Experimental Setup

The experiments were conducted in a simulated environment using the PyBullet physics engine. The UR3e robot was modeled using the URDF format, and the simulation environment was designed to closely resemble the real-world setup. The training process was implemented using the Stable-Baselines3 framework, with the SAC algorithm selected for its efficiency and stability in high-dimensional continuous action spaces.

### 4.2. Training Performance of the DRL Policy

In this subsection, we present the training performance of the DRL policy over the course of 6 million steps. The training process was monitored using various metrics, including the average reward, success rate, and episode length.

#### 4.2.1. Success Rate

**Figure 5:**
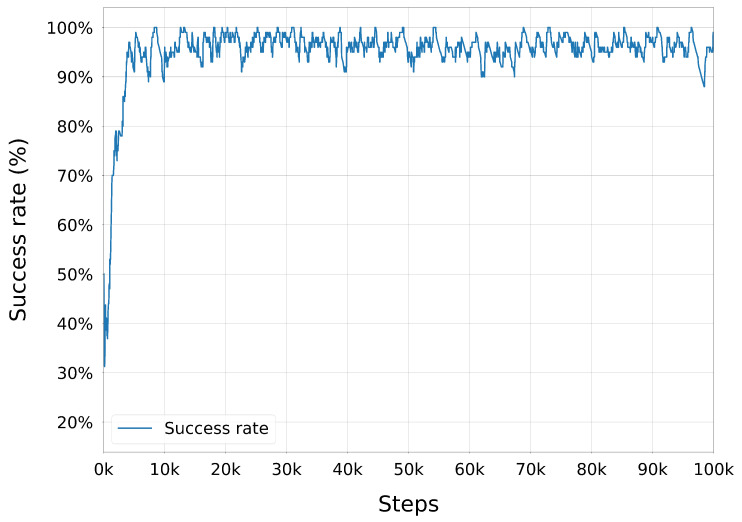
Training performance of the DRL policy success rate over the initial training steps.

Figure 5 illustrates the percentage of successful episodes over the total number of episodes during the first 100,000 steps. Initially, the performance is low due to the agent’s exploration phase, where expert demonstrations are not yet fully integrated into the training process. However, as the agent learns from these trajectories and refines its policy, the percentage of successful episodes improves significantly.

**Figure 6:**
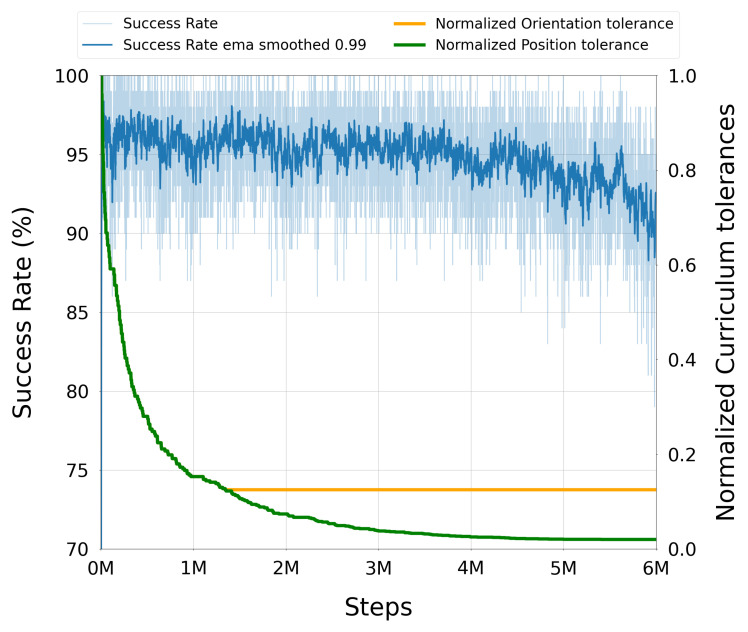
Training performance of the DRL policy, illustrating the success rate progression over the entire training process, highlighting the agent’s learning curve and adaptation to increasingly challenging goals.

Figure 6 shows the success rate over the entire training process. The success rate remains higher than 90% after the first 10,000 steps, but as the curricula progress and the goals become more challenging, the success rate slightly fluctuates. The agent learns to adapt to the new goals and improve its performance over time. The success rate reaches a plateau after approximately 6 million steps, indicating that the agent has learned to reach the goal pose effectively across a wide range of configurations.

#### 4.2.2. Reward

**Figure 7:**
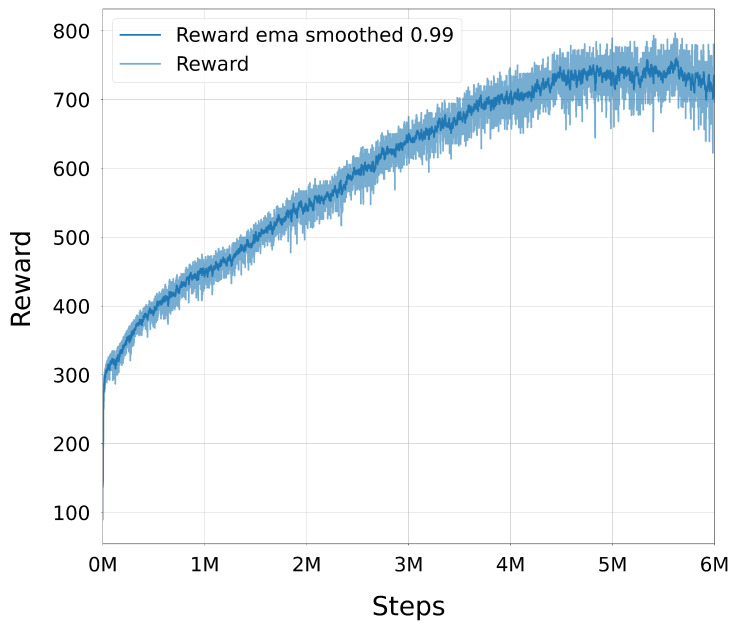
Training performance of the DRL policy, showing the success rate progression over training steps, highlighting the agent’s learning curve and adaptation to the task.

Figure 7 shows the reward progression over the course of the training process. The reward is computed as the mean of the rewards obtained during each episode. It starts at a low value and gradually increases as the agent learns to reach the goal pose more effectively. With the integration of expert demonstrations, the reward increases significantly during the first 10,000 steps. The agent learns to reach the goal pose more effectively, resulting in higher rewards. As the training progresses, the reward continues to improve as the curriculum becomes more challenging, and the reward function is adjusted to reflect the new tolerances. The reward reaches a plateau after approximately 6 million steps, indicating that the agent has learned to reach the goal pose effectively up to the specified tolerances.

#### 4.2.3. Curriculum Progression

**Figure 8:**
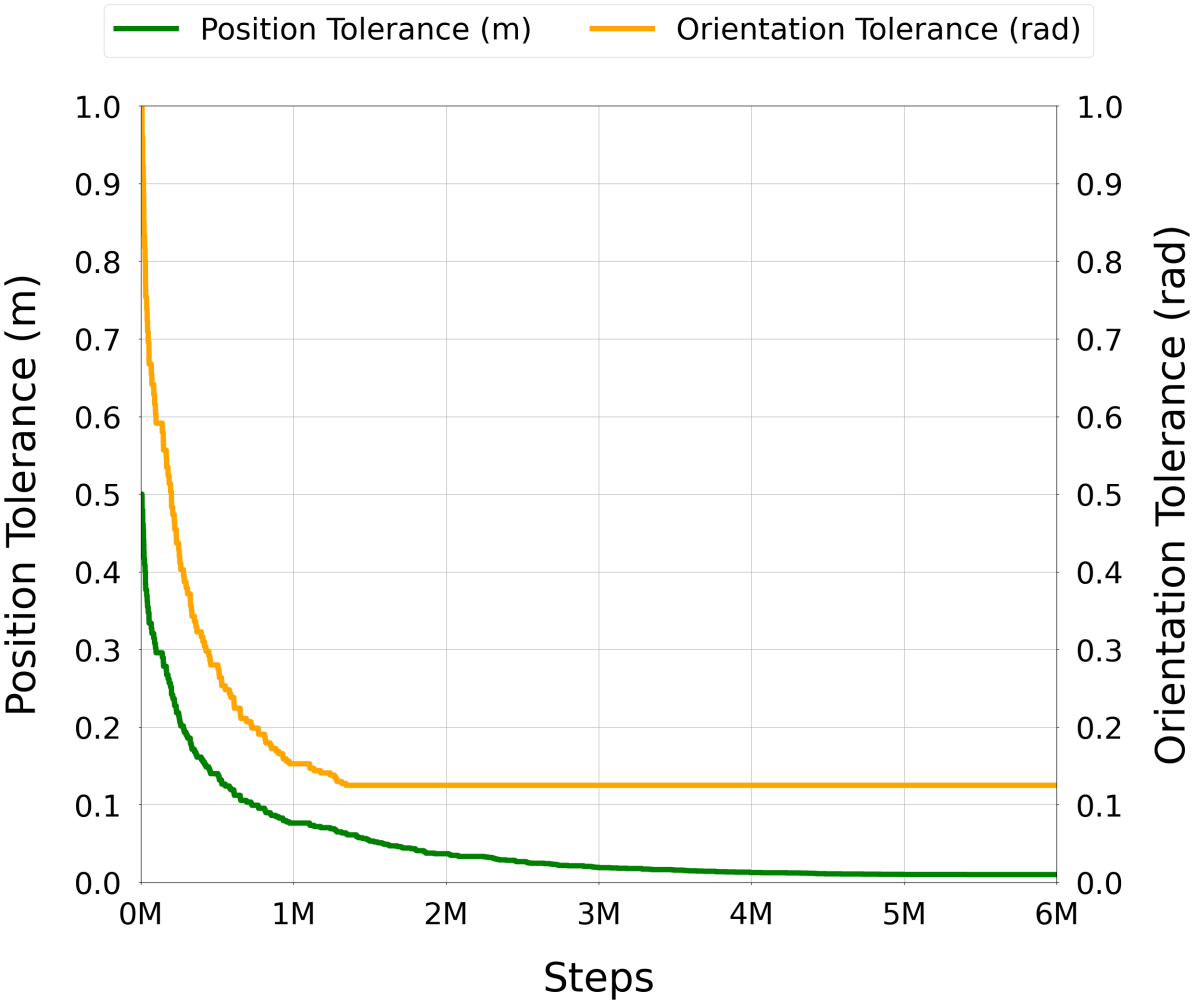
Curriculum-level progression for the robot end-effector, showing the gradual tightening of tolerances for both position and orientation.

Figure 8 shows the goal position and orientation tolerances over the course of the training process. It illustrates the progression of the curriculum levels, with the position and orientation tolerances decreasing as the agent learns to reach the goal poses. The agent starts with relaxed tolerances and gradually tightens them as it learns to reach the goal poses more accurately. Initially, the progress is fast as the agent learns to reach the goal poses with relaxed tolerances. As the training progresses, the agent encounters more challenging tolerances, leading to a slower progression in the curriculum levels. The figures show that the agent successfully adapts to the new tolerances and improves its performance over time. It is important to note that the curriculum levels are coupled for position and orientation, preventing the agent from focusing solely on one aspect of the task. This coupling ensures that the agent learns to reach the goal poses in both position and orientation simultaneously, which is crucial for effective motion planning in robotic applications. The figures show that for the agent, it is harder to reach the position than the orientation. This is evident from the slower progression in the position curriculum levels compared to the orientation curriculum levels. The agent requires more training steps to achieve tighter tolerances in position, indicating that the translational component of the task poses a greater challenge than the rotational component, especially for the higher curriculum levels.

#### 4.2.4. Episode Length

**Figure 9:**
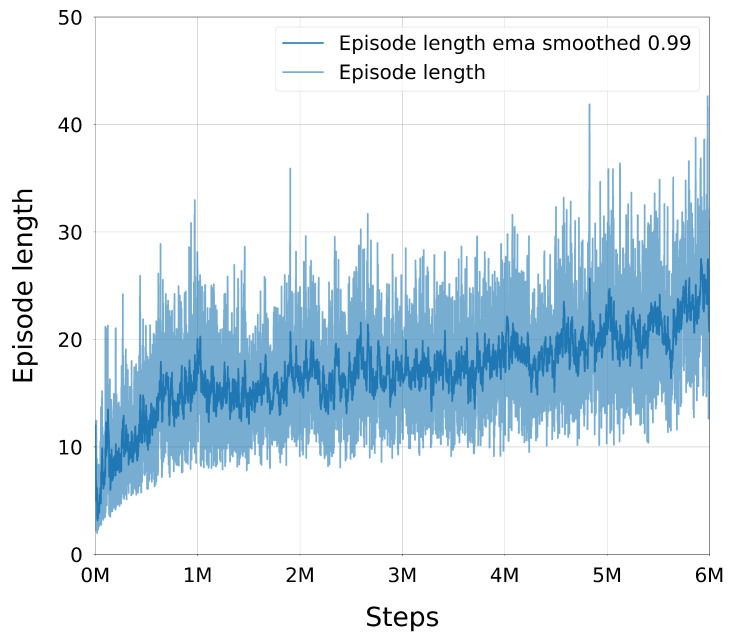
Average episode length of the DRL policy during training, illustrating the progression and stabilization of the agent’s performance over training steps.

Figure 9 shows the average episode length over the course of the training process. The average episode length is computed as the mean number of steps taken to reach a termination condition. The initial low episode length is due to the agent’s exploration phase, where the expert demonstrations are not yet fully integrated into the training process. In this phase, the agent provokes collisions and fails to reach the goal pose, leading to shorter episode lengths. As the agent learns from the expert trajectories and refines its policy, the episode length increases until it reaches a plateau. The episode length remains relatively stable after approximately 6 million steps with minor increases, indicating that, as the goals become more challenging, more steps are required to reach the goal pose. The agent learns to adapt to the new goals and improve its performance over time.

### 4.3. Comparison Between DRL and OMPL-Based Planners

To assess the relative performance of the proposed DRL-based planner and classical sampling-based motion planners from MoveIt’s OMPL pipeline, a systematic comparison was carried out across the common dataset of goal pairs. This comparison uses the test set of 10,429 trajectories, which were not used during training or validation. All planners were tested under identical workspace conditions, and the resulting trajectories were analyzed according to the following key performance metrics:

- Success rate: percentage of planning requests that resulted in valid, collision-free paths.
- Planning time: time required to compute a trajectory (DRL includes inference and TOPPRA time parameterization; OMPL includes full MoveIt pipeline).
- Trajectory length: total number of waypoints in the path (pre-parameterization).

#### 4.3.1. Overall Performance

Table 3 presents the success rate and the average and standard deviation of key performance metrics for the evaluated planners across the validation dataset, including success rate, planning time, and trajectory length. These metrics provide a high-level summary of the comparative performance.

The smoothness of the joint-space trajectories is computed as the same quadratic objective formulation employed in TOPPRA. In this context, the trajectory is represented as $q\left(t\right)\in R^{n}$, where *n* is the number of joints. The smoothness metric *S* is defined as the integral of the squared joint acceleration norm over the execution time,

(2) $$S\;=\;\int_{t_{0}}^{t_{f}}{∥\ddot{q}\left(t\right)∥}_{2}^{2}\;dt,$$

where ${∥\cdot ∥}_{2}$ denotes the Euclidean norm across all joints. This metric penalizes rapid changes in velocity, effectively favoring trajectories with lower acceleration magnitudes over time. In discrete form, with sampled accelerations $\ddot{q}\left[k\right]$ at times $t_{k}$, the integral is approximated using the trapezoidal rule as

(3) $$S\;\approx \;\sum_{k=1}^{N}{∥\ddot{q}\left[k\right]∥}_{2}^{2}\;\Delta t_{k},$$

where $\Delta t_{k}=t_{k}-t_{k-1}$. This formulation is directly aligned with the convex quadratic cost functions supported by TOPPRA, which allow optimizing not only for traversal time but also for motion smoothness [72].

Detailed box plots are provided for each metric. These box plots exclude outliers to focus on the central distribution of results. Only the most relevant planners are shown, as some planners exhibited results with metrics far from meaningful values, making the plots unbalanced for comparison.

#### 4.3.2. Planning Time and Waypoint Complexity

**Figure 10:**
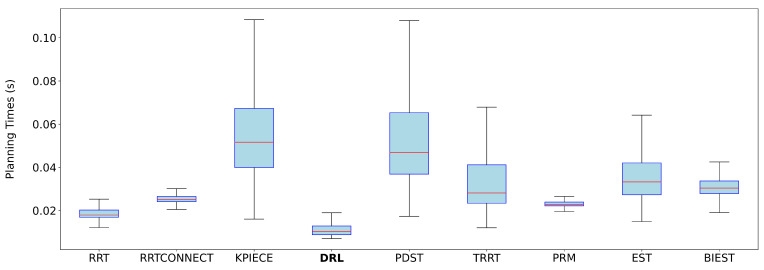
Box plot illustrating the planning time (in seconds) for each method. The DRL-based planner demonstrates significantly faster and more consistent inference times compared to OMPL planners. Note that only successful cases are represented, and outliers (flyers) have been omitted for clarity.

Figure 10 presents the distribution of planning times for each method. As expected, the DRL policy produces deterministic inference results with low variance and significantly reduced planning time. OMPL planners exhibit much higher variance, with some instances exceeding several seconds due to roadmap construction or constraint resolution. It is important to note that for every failed planning attempt, the OMPL planning pipeline exhausted the maximum number of attempts, leading to a planning time of 5 s. This is omitted from the box plot for clarity.

**Figure 11:**
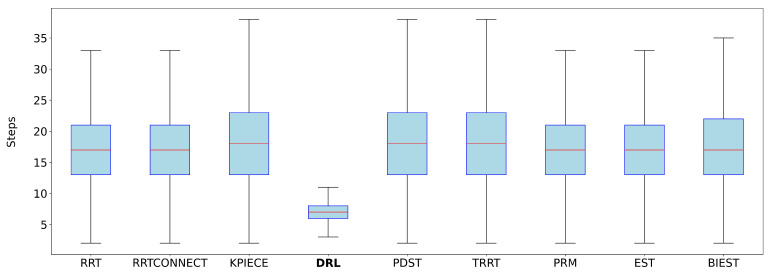
Box plot illustrating the number of waypoints per planned trajectory across different methods. DRL-generated paths are notably more compact and efficient compared to those produced by OMPL planners.

Figure 11 shows the number of waypoints per path. The DRL policy produces smoother, more direct paths with fewer discrete steps compared to OMPL-generated trajectories, which tend to be longer due to sampling density and post-processing limitations.

#### 4.3.3. Configuration Space Path Length

**Figure 12:**
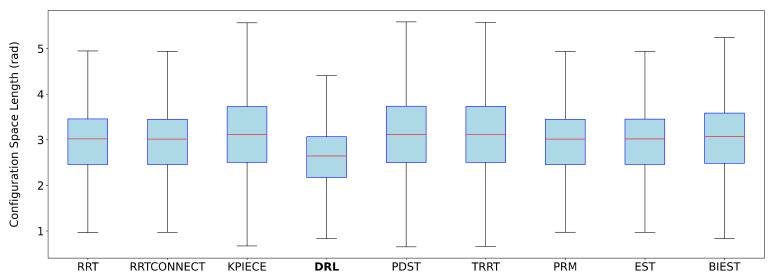
Box plot illustrating the configuration space path lengths (joint-space distances) for planned trajectories across different methods. Shorter path lengths indicate more efficient joint-space motion.

Figure 12 shows the distribution of the total path length in configuration space, computed as the cumulative Euclidean distance between consecutive joint-space waypoints. This metric offers a more detailed insight into the overall motion efficiency. The DRL policy consistently produces shorter joint-space paths, likely due to its incremental control strategy and its tendency to avoid redundant motions.

#### 4.3.4. Failure Modes and Comparison

Across the 10,429-query test set, our SAC-based planner failed to produce collision-free trajectories in approximately 5.9% of cases. These failures predominantly occur for goal poses located near the extreme boundaries of the UR3e’s workspace or due to high-precision goal tasks at the tightest curriculum tolerances. In these scenarios, the policy occasionally oscillates or cannot satisfy both position and orientation constraints within the allotted 200 steps.

The observed failures at the edges of the workspace can be linked to both task-specific and algorithmic factors. From a robotics perspective, extreme poses often lie near the manipulator’s kinematic limits, where feasible solution regions are narrow and discontinuous.

From a deep reinforcement learning perspective, such boundary states are typically underrepresented in the training data distribution. Even with uniform workspace sampling, collision filtering and the curriculum’s gradual tolerance tightening reduce the relative frequency of these extreme cases in early training stages. Consequently, the policy’s state–action value function is less accurate in these regions. Furthermore, reward shaping based primarily on Euclidean pose errors can lead to gradient disappearance near boundaries if the agent’s actions produce negligible improvements due to physical constraints, resulting in weak learning signals. These factors combine to create conditions in which the policy generalizes poorly to the workspace edges, despite strong performance in the interior.

Mitigation strategies include targeted oversampling of boundary poses, curriculum phases explicitly dedicated to extreme regions, or auxiliary exploration bonuses to increase coverage. Future work may also explore adaptive reward scaling near kinematic limits to maintain informative gradients for policy improvement.

By contrast, classical sampling-based planners such as RRTConnect (approximately 7.1% failure rate) can sometimes succeed on these boundary cases through exhaustive sampling, though at the cost of planning times up to several seconds. Conversely, the DRL planner successfully handles certain narrow-passage queries—where OMPL planners fail repeatedly—by leveraging its learned collision-avoidance priors. This complementary behavior suggests that incorporating fallback classical planning for the DRL’s rare failure cases (and vice versa) could yield a more robust hybrid system.

## 5. Discussion

### 5.1. Advantages of DRL for Industrial Motion Planning

The experimental results demonstrate clear advantages of the DRL-based planner over traditional sampling-based motion planning techniques when applied to the task of collision-free goal reaching in the UR3e robot’s workspace. The most prominent benefit is the significantly reduced planning time: the DRL policy consistently computes valid actions within milliseconds, in stark contrast to OMPL planners that often require hundreds of milliseconds to several seconds. This determinism and low-latency response make the DRL approach suitable for real-time applications, including moving target poses, where responsiveness is critical.

Another major advantage lies in trajectory compactness. DRL-generated paths exhibit fewer waypoints and shorter configuration space lengths. This behavior can be attributed to the continuous control formulation of the policy and the curriculum-based reward shaping, which encourages direct and minimal joint-space movement. In contrast, OMPL planners, being sampling-based, rely on a fixed sampling density and may produce longer paths with more waypoints due to their exploration strategies. This results in smoother trajectories that are easier to execute on real hardware, reducing wear and tear on the robot’s joints and improving overall efficiency.

The DRL planner also achieved a high success rate, slightly outperforming most of the OMPL planners in the same benchmark conditions. This indicates that the agent successfully generalized within the sampled workspace and learned robust collision-free behaviors. The use of curriculum learning contributed to this result by enabling the agent to first solve easy instances and progressively adapt to tighter tolerances. The success rate remained stable above 90% across a wide range of configurations, even when the curriculum posed increasingly strict constraints.

The deterministic nature of DRL also eliminates the variability observed in OMPL-based approaches. Since both the trained policy and the post-processing step (TOPPRA) are deterministic, the only source of uncertainty is the number of steps needed to reach the goal, which was shown to be consistently bounded across tasks. This provides strong guarantees on worst-case behavior, which is often difficult to ensure in sampling-based planning. The use of expert demonstrations from OMPL planning pipeline trajectories significantly accelerated training and improved sample efficiency. This hybrid approach helped bootstrap the learning process, allowing the agent to benefit from classical planning knowledge without inheriting its runtime constraints. This synergy highlights a promising direction where classical methods can be used as priors to warm-start learning-based planners for high-performance industrial deployment.

Finally, the use of the same ROS interfaces and MoveIt! pipeline for both the DRL-based planner and OMPL planners allows for seamless integration into existing robotic systems. This compatibility facilitates the deployment of the DRL policy in real-world applications without requiring modifications to the software architecture.

### 5.2. Limitations and Failure Cases

Despite the promising results obtained with the DRL-based planner, several limitations must be acknowledged. Failure cases were also observed during the early phases of training, where the policy frequently failed due to collisions or an inability to make progress toward the goal. While expert demonstrations mitigated this issue, their usefulness depends on the quality and diversity of the dataset, and integrating such demonstrations requires a well-structured replay buffer and careful management of exploration vs. imitation. Moreover, the DRL training process remains computationally expensive, requiring several million environment steps to converge (approximately 22 h). This training burden contrasts sharply with the plug-and-play nature of classical OMPL planners, which require no learning phase and can be deployed immediately.

Another important consideration is the potential advantage of multi-query planners in static environments. Multi-query planners, such as PRM or LazyPRM, are designed to handle multiple planning queries efficiently by constructing a reusable roadmap of the configuration space. Once the roadmap is built, these planners can quickly find paths for new start and goal configurations by leveraging the precomputed graph structure. This makes them particularly effective in scenarios where the environment remains static and multiple planning requests are expected. However, the upfront computational cost of building the roadmap can be significant, and the quality of the roadmap heavily depends on its density and coverage of the configuration space.

Transfer learning [73] emerges as a powerful solution to address both the computational cost of training and the adaptability to new environments. By leveraging knowledge from previously trained policies, transfer learning can significantly reduce the number of training steps required to adapt to unseen environments or tasks. This capability not only mitigates the initial training burden but also ensures that the DRL-based planner remains versatile and scalable, making it a compelling choice for industrial applications where environments may evolve over time or require frequent reconfiguration.

### 5.3. Training Overhead and Industrial Deployment Considerations

While the DRL-based planner demonstrates superior planning speed and trajectory compactness, it requires significant upfront computational resources for training.

In contrast, classical OMPL planners are plug-and-play, requiring no training and minimal configuration effort, making them highly suitable for rapid deployment and integration.

To better understand the trade-offs, Table 4 compares the overall computational costs of both approaches. The DRL planner amortizes its training cost across thousands of inferences, yielding deterministic sub-10 ms planning latency once trained. OMPL planners, on the other hand, exhibit variable planning times across queries, often exceeding hundreds of milliseconds or seconds in complex workspaces.

While the upfront cost of DRL is non-negligible, this investment can be justified in settings such as the following:

- The same planning task is repeated across many cycles (e.g., 24/7 manufacturing).
- Real-time responsiveness is critical.
- Motion quality and energy efficiency are prioritized.

Additionally, first experiments indicate that simple retrainings on new datasets can be performed in significantly less time than the initial training, depending on the complexity of the new environment. This makes the DRL approach increasingly attractive for dynamic or reconfigurable industrial applications.

### 5.4. Comparison to Sampling-Based Planning

The comparative analysis between the DRL policy and MoveIt’s OMPL planners highlights complementary strengths of each approach, offering insights into when one may be favored over the other.

Classical sampling-based planners, such as RRT, PRM, or KPIECE, exhibit a high degree of generality and are well-suited for environments with changing constraints, varying kinematic chains, or unknown obstacles. Their flexibility and modularity make them ideal for prototyping in diverse robotic systems, and they can operate immediately without any prior data or learning phase. Moreover, OMPL planners natively handle motion constraints, including joint limits and collision avoidance, via the MoveIt planning pipeline, making them robust for a wide range of planning tasks.

However, these benefits come at the cost of computational overhead and non-deterministic behavior. Planning times are highly variable depending on the specific planner, goal pair, and search space complexity. In contrast, the DRL policy generates motions with near-constant latency and complete determinism once trained. This enables seamless integration into real-time control loops, a requirement in many industrial applications where planning speed and predictability are critical.

Another key difference lies in trajectory quality. DRL-generated paths are significantly shorter in both the number of waypoints and configuration space length. This suggests more direct and efficient joint-space motion, which is likely a result of policy optimization for task completion rather than exploration. While OMPL planners employ smoothing and shortcutting, they remain constrained by discrete sampling resolution and the inherent randomness of the roadmap or tree construction process.

The success of the DRL approach depends on prior training on a representative dataset. This makes it less adaptable to novel or unseen constraints without additional learning. In contrast, OMPL planners are stateless and can adapt to arbitrary start and goal poses within the robot’s kinematic limits, making them more versatile for exploration or dynamic environments.

### 5.5. Future Work

Future work will focus on three main directions. First, we aim to explore the application of transfer learning to adapt the trained DRL policies to new scenarios and environments. In particular, we will study how the planner generalizes to irregular and curved workspace boundaries, as opposed to the planar configurations considered in this work. While the present dataset was limited to the robot’s reachable region with the floor plane as the main obstacle, future experiments will test adaptability to more complex environments with variable obstacle geometries. This approach will leverage the knowledge acquired during the initial training phase to significantly reduce the computational cost and time required for retraining in novel settings. Transfer learning is particularly promising for dynamic or reconfigurable industrial environments, where the robot’s workspace or task requirements may change frequently. By fine-tuning the policy on new datasets or using domain adaptation techniques, we aim to extend the generalization capabilities of the DRL planner while maintaining its low-latency and deterministic performance. This will also allow us to evaluate the policy’s ability to adapt rapidly to new, highly variable environments and task configurations, thereby extending beyond the controlled benchmark presented here.

Second, we plan to analyze and benchmark the use of DRL policies as a preprocessor for optimization-based motion planning algorithms, such as CHOMP and STOMP. These algorithms excel at refining trajectories to meet additional constraints, such as smoothness, obstacle clearance, and dynamic feasibility, but often require a high-quality initial trajectory to converge efficiently. By providing a near-optimal initialization, the DRL planner can significantly reduce the computational burden of these optimization-based methods, enabling faster convergence and higher-quality solutions. While in this work we did not implement a hybrid planner, preliminary results suggest that this hybrid approach could combine the strengths of both paradigms, offering the speed and determinism of DRL with the precision and constraint-handling capabilities of optimization-based planners. Future work will include systematic benchmarking of this hybrid framework to evaluate its performance across diverse industrial scenarios.

Lastly, we will pursue hardware validation on the UR3e and generalization to more complex robotic systems. At this stage, we will also incorporate sensor noise and small disturbances in the experimental evaluation, as robustness to such factors is essential for bridging the sim-to-real gap. Although noise does not directly affect execution in our current setup, since complete trajectories are generated offline, its effect on state estimation will be systematically studied in the hardware validation phase.Because our policy runs entirely offline (requiring only the robot’s kinematic model, an initial joint configuration, and a target end-effector pose), it is expected to transfer to the physical UR3e nearly plug-and-play; we will validate this and apply lightweight calibration or fine-tuning on a small set of real trajectories if needed to correct any residual kinematic offsets. The same dataset generation and training pipeline can be directly extended to dual-arm manipulators by expanding the state/action spaces and encoding coordination constraints, just as OMPL planners do. Multi-agent systems stand to benefit from the planner’s low-latency inference, enabling real-time replanning for collision avoidance among agents. Furthermore, trajectory execution will follow a two-step workflow: first, validation in simulation to ensure the correctness of the planned path, and second, execution on the real robot through standard ROS interfaces. This approach leverages existing industrial safety mechanisms while providing an additional layer of verification, thereby ensuring safe and reliable operation of the DRL-generated trajectories in real industrial lines.

## 6. Conclusions

This work presents a comprehensive comparison between classical MoveIt’s OMPL sampling-based motion planning pipeline and a DRL approach based on SAC for industrial robot motion planning. The study focused on the UR3e robotic arm equipped with an RG2 gripper in a workspace constrained only by self-collisions and ground contact, allowing for a controlled yet representative evaluation of planning performance.

To support this comparison, a large-scale dataset was generated using IK sampling and filtering, followed by motion planning using MoveIt-integrated OMPL planners. A DRL policy was trained using curriculum learning and expert demonstrations extracted from the same OMPL-generated trajectories. The trained DRL policy was then integrated with TOPPRA for time-optimal parameterization and compared against traditional planners across several metrics.

The results demonstrate that the DRL-based planner achieves competitive or superior performance in terms of success rate, planning time, trajectory compactness, and configuration space length. Inference using the trained policy is not only deterministic and fast but also produces smoother and shorter joint-space paths. These characteristics make it particularly well-suited for time-sensitive industrial applications.

Nonetheless, the DRL policy exhibits limitations when it comes to handling high-precision goals, especially in the translational domain, and its applicability is currently confined to simulation environments. Although the training environment closely matches the UR3e’s kinematics and collision model, real-world deployment would introduce unmodeled dynamics, sensor noise, and compliance effects. These aspects were not tested in this study, limiting conclusions about physical robustness. Future work will focus on hardware validation and sim-to-real techniques to bridge this gap. OMPL planners, in contrast, retain advantages in adaptability and generality for new tasks and environments, albeit at a higher computational cost.

The findings suggest that DRL, when augmented with structured training techniques and expert knowledge, can serve as a high-performance alternative to traditional motion planners in deterministic and repetitive industrial contexts. Moreover, DRL demonstrates exceptional potential when combined with classical methods, effectively addressing the cold-start problem in highly complex environments or near-infinite workspaces. This synergy leverages the strengths of both paradigms, enabling robust and efficient motion planning even in challenging scenarios.

While hardware validation on the UR3e remains as future work, the use of an identical MoveIt! planning stack and conservative TOPPRA parameterization provides strong confidence that the observed simulation-based performance will translate effectively to real industrial settings.

To summarize, this work presents the first large-scale comparison of DRL (SAC) and sampling-based planners for 6-DoF industrial manipulators, leveraging over 100,000 expert trajectories, curriculum learning, and expert-demonstration bootstrapping to achieve sub-10 ms deterministic planning with >94% success. Open questions remain, including the energy efficiency of DRL-generated trajectories compared to classical planners and the balance between training cost and long-term operational savings.

## Figures and Tables

**Table 1:** Final SAC training hyperparameters.

Parameter	Value
Learning rate	$3\times 10^{-4}$
Batch size	4096
Replay buffer size	$10^{6}$
Discount factor (γ)	0.99

**Table 2:** Hardware specifications used for experiments.

Component	Specification
CPU	Intel Core i7-10700F @ 2.90 GHz
RAM	16 GiB DDR4 @ 4400 MHz
GPU	NVIDIA GeForce RTX 2060
SSD	Samsung 870 1 TB

**Table 3:** Summary of averaged performance metrics for DRL and OMPL planners. The table highlights key metrics, including planning time, waypoint count, success rate, and maximum planning time. For planning time and waypoint count, the average values along with their standard deviations are provided to reflect variability across trials. The best performance for each metric is highlighted in bold.

Planner	Planning Time (s)	Waypoint Number	Success Rate (%)	Maximum Planning Time (s)	Smoothness
**DRL**	**0.01322 ± 0.003**	**7.20 ± 4.25**	**94.12**	**0.023**	**1.2286 ± 0.4285**
BITSTAR	0.03349 ± 0.190	18.46 ± 10.54	92.91	5.028	1.2869 ± 0.5108
PRM	0.03360 ± 0.182	18.61 ± 11.09	92.97	5.039	1.2987 ± 0.5149
RRTConnect	0.03542 ± 0.203	18.51 ± 10.61	92.86	5.048	1.2874 ± 0.4983
PROJEST	0.03665 ± 0.067	19.01 ± 12.07	92.60	5.016	1.2941 ± 0.5124
EST	0.03992 ± 0.065	18.87 ± 11.92	92.86	5.013	1.2961 ± 0.5141
BIEST	0.04051 ± 0.185	19.78 ± 12.45	91.36	5.042	1.3177 ± 0.5372
BITRRT	0.04057 ± 0.278	19.29 ± 13.34	91.68	5.043	1.3189 ± 0.5353
RRT	0.04578 ± 0.236	18.56 ± 10.83	92.92	5.052	1.2999 ± 0.5251
KPIECE	0.07106 ± 0.220	20.56 ± 13.03	90.59	5.233	1.3478 ± 0.5485
STRIDE	0.07476 ± 0.176	20.75 ± 13.75	90.58	5.054	1.3475 ± 0.5691
PDST	0.11133 ± 0.339	20.32 ± 12.33	90.49	5.067	1.3372 ± 0.5289
TRRT	0.28084 ± 0.963	20.33 ± 12.04	90.62	5.085	1.3572 ± 0.5793
BKPIECE	0.38242 ± 0.265	18.79 ± 11.65	92.74	5.046	1.2923 ± 0.5306
SBL	0.52241 ± 0.296	18.85 ± 12.04	92.95	5.055	1.3016 ± 0.5466
LBKPIECE	1.44631 ± 0.534	20.87 ± 13.79	90.47	5.121	1.3627 ± 0.6179
LAZYPRM*	5.00021 ± 0.002	18.37 ± 11.65	92.46	5.074	1.2799 ± 0.4776
RRT*	5.003 ± 0.006	18.08 ± 9.38	92.99	5.008	1.2713 ± 0.4597
PRM*	5.00617 ± 0.005	18.14 ± 9.67	92.94	5.954	1.2880 ± 0.4979

**Table 4:** Comparison of training and inference costs for DRL and OMPL.

Metric	DRL (SAC)	Sampling Based
Training time	22 h (one-time)	None
Hardware required	GPU (RTX 2060)	CPU-only
Per-query planning time	∼13 ms	50 ms–5 s
Success rate (test set)	94.1%	90–93%
Trajectory compactness	7 points avg.	18–20 points avg.
Adaptability to new setups	Retraining needed	Direct use

## Data Availability

Data are contained within the article.
